# Effects of community-based antiretroviral therapy initiation models on HIV treatment outcomes: A systematic review and meta-analysis

**DOI:** 10.1371/journal.pmed.1003646

**Published:** 2021-05-28

**Authors:** Ingrid Eshun-Wilson, Ajibola A. Awotiwon, Ashley Germann, Sophia A. Amankwaa, Nathan Ford, Sheree Schwartz, Stefan Baral, Elvin H. Geng

**Affiliations:** 1 Division of Infectious Diseases, Washington University School of Medicine, Washington University in St. Louis, Saint Louis, Missouri, United States of America; 2 Division of Epidemiology and Biostatistics, Faculty of Medicine and Health Sciences, Stellenbosch University, Stellenbosch, South Africa; 3 Knowledge Translation Unit, University of Cape Town Lung Institute, Cape Town, South Africa; 4 Johns Hopkins Bloomberg School of Public Health, Baltimore, Maryland, United States of America; 5 Global Hepatitis Programme, Department of HIV/AIDS, World Health Organization, Geneva, Switzerland; Boston University School of Public Health, UNITED STATES

## Abstract

**Background:**

Antiretroviral therapy (ART) initiation in the community and outside of a traditional health facility has the potential to improve linkage to ART, decongest health facilities, and minimize structural barriers to attending HIV services among people living with HIV (PLWH). We conducted a systematic review and meta-analysis to determine the effect of offering ART initiation in the community on HIV treatment outcomes.

**Methods and findings:**

We searched databases between 1 January 2013 and 22 February 2021 to identify randomized controlled trials (RCTs) and observational studies that compared offering ART initiation in a community setting to offering ART initiation in a traditional health facility or alternative community setting. We assessed risk of bias, reporting of implementation outcomes, and real-world relevance and used Mantel–Haenszel methods to generate pooled risk ratios (RRs) and risk differences (RDs) with 95% confidence intervals. We evaluated heterogeneity qualitatively and quantitatively and used GRADE to evaluate overall evidence certainty. Searches yielded 4,035 records, resulting in 8 included studies—4 RCTs and 4 observational studies—conducted in Lesotho, South Africa, Nigeria, Uganda, Malawi, Tanzania, and Haiti—a total of 11,196 PLWH. Five studies were conducted in general HIV populations, 2 in key populations, and 1 in adolescents. Community ART initiation strategies included community-based HIV testing coupled with ART initiation at home or at community venues; 5 studies maintained ART refills in the community, and 4 provided refills at the health facility. All studies were pragmatic, but in most cases provided additional resources. Few studies reported on implementation outcomes. All studies showed higher ART uptake in community initiation arms compared to facility initiation and refill arms (standard of care) (RR 1.73, 95% CI 1.22 to 2.45; RD 30%, 95% CI 10% to 50%; 5 studies). Retention (RR 1.43, 95% CI 1.32 to 1.54; RD 19%, 95% CI 11% to 28%; 4 studies) and viral suppression (RR 1.31, 95% CI 1.15 to 1.49; RD 15%, 95% CI 10% to 21%; 3 studies) at 12 months were also higher in the community-based ART initiation arms. Improved uptake, retention, and viral suppression with community ART initiation were seen across population subgroups—including men, adolescents, and key populations. One study reported no difference in retention and viral suppression at 2 years. There were limited data on adherence and mortality. Social harms and adverse events appeared to be minimal and similar between community ART initiation and standard of care. One study compared ART refill strategies following community ART initiation (community versus facility refills) and found no difference in viral suppression (RD −7%, 95% CI −19% to 6%) or retention at 12 months (RD −12%, 95% CI −23% to 0.3%). This systematic review was limited by few studies for inclusion, poor-quality observational data, and short-term outcomes.

**Conclusions:**

Based on data from a limited set of studies, community ART initiation appears to result in higher ART uptake, retention, and viral suppression at 1 year compared to facility-based ART initiation. Implementation on a wider scale necessitates broader exploration of costs, logistics, and acceptability by providers and PLWH to ensure that these effects are reproducible when delivered at scale, in different contexts, and over time.

## Abstract 2

Ingrid Eshun-Wilson and co-workers assess the available evidence on community-based treatment initiation for people with HIV.

## Author summary

**Why was this study done?:**

- Over the last decade HIV services have increasingly moved out of the health facility and into the community through the provision of decentralized and differentiated HIV care.
- It remains unclear however whether initiating ART in a community setting will result in treatment and safety outcomes that are comparable to facility-based ART initiation.

**What did the researchers do and find?:**

- We conducted a systematic review to identify studies where ART was initiated at community locations, including homes, mobile vans, or other community venues.
- We identified 8 studies (including 11,196 HIV-positive people), 7 of which were conducted in sub-Saharan Africa; 4 were randomized controlled trials and 4 were cohort studies.
- The methodological quality of the randomized controlled trials was high, but cohort data were of poorer quality. Studies were generally pragmatic in design, but implementation outcomes were infrequently reported.
- Based on meta-analysis of this limited dataset, it appeared that ART initiation in the community resulted in higher ART uptake, higher retention, and greater viral suppression at 1 year compared to facility-based ART initiation and refill, among HIV-positive people offered ART. These findings were consistent across population subgroups and various implementation strategies.
- There were insufficient data on serious adverse events or mortality to draw firm conclusions on these outcomes.

**What do these findings mean?:**

- Community ART initiation may result in better outcomes than ART initiation in traditional health facilities.
- To increase the robustness of these findings, high-quality implementation research conducted in diverse settings, exploring optimum combinations of community ART initiation and ART refill strategies over longer time periods, will be critical.

## Introduction

Initiating antiretroviral therapy (ART) in the community setting and outside of traditional health facilities represents an innovative addition to “differentiated service delivery” models, which seek to offer a greater range of options that meet a diversity of patient needs in the global HIV service delivery enterprise. Community-based ART initiation has several potential benefits including reducing psychological and structural barriers that newly diagnosed HIV-positive people face in order to access a facility, as well as further decongesting crowded facilities themselves. With a shift over the last decade to rapidly initiating ART in those who test HIV-positive, and decentralizing and differentiating follow-up after treatment initiation in the facility, community ART initiation represents the next step toward more patient-centered services and may bridge the critical gap between testing and linkage to ART—a point in the HIV care cascade when many disengage from care [1–3].

Despite this rationale, few studies have explored the effect of community ART initiation on either short- or long-term outcomes. Community-based HIV testing, including mobile testing, self-testing, testing campaigns, workplace testing, and index testing, frequently shows higher coverage and uptake than traditional facility-based testing and has the ability to reach those underserved by routine facility-based testing, particularly men and key populations [4,5]. Community-based medication refill for patients stable on ART, such as distribution directly to patients’ homes or to community pick-up locations and pharmacies, has generally demonstrated success [6,7]. ART initiation, however, has traditionally been reserved for facilities because of the perceived intensity of the encounter, but this assumption is not empirically supported. Qualitative data suggest that the act of going to a clinic is intimidating and confusing, particularly in environments where stigma is present—making community ART initiation a potentially important innovation [8].

A number of studies examining the effects of community ART initiation have been conducted, but synthesis and review are needed to appraise the quality of the data as well as assess the top-line evidence of the effect of this approach on immediate and medium-term outcomes. In addition, systematic reviews are required as a part of the guideline development processes led by the HIV department at the World Health Organization (WHO), and therefore an important step in the translation of evidence to practice. To explore the effect of initiating ART in a community setting on HIV treatment outcomes, we conducted a systematic review that additionally characterized features of community ART initiation strategies to inform policy and implementation.

## Methods

### Search strategy and selection criteria

The protocol for this systematic review was registered on PROSPERO (CRD42019130272) and followed PRISMA guidelines [9,10] (S1 Table). We searched the Cochrane Central Register of Controlled Trials (CENTRAL, published in the Cochrane Library), MEDLINE (PubMed), Embase (OVID), Africa-Wide Information and CINAHL (EBSCOhost), LILACS, and Web of Science Core Collection from 1 January 2013 until 15 April 2019; this start date was based on the 2013 WHO recommendation for decentralization of HIV treatment and care [11], and a previous systematic review that searched for studies published from 1996 to 2013 and found no published studies on community-based ART initiation prior to 2013 [12] (S1 Appendix). Searches were updated on 1 April 2020 and again on 22 February 2021.

We included randomized and non-randomized study designs that enrolled HIV-positive people of any age, conducted in low- and middle-income countries, and compared community ART initiation to facility-based ART initiation or to another community-based ART initiation strategy. We defined community ART initiation as initiation of ART outside of a traditional health facility or workplace health center, by any cadre of health staff. Community settings included, for example, mobile health services, community centers, and patients’ homes. ART could be offered and initiated in the community and subsequently maintained in the community or at a health center. Our comparison arm was ART initiation in a traditional health facility; ART maintenance after initiation could occur within or outside of the health facility. No language or age restrictions were applied to the search. We additionally searched HIV/AIDS conferences including International AIDS Society (IAS) conferences and the Conference on Retroviruses and Opportunistic Infections (CROI) until 10 March 2021, as well as the reference lists of included studies and relevant systematic reviews. We also searched ClinicalTrials.gov and the WHO International Clinical Trials Registry Platform for ongoing studies.

### Data extraction and methodological quality assessment

Abstract and full-text screening was done in duplicate, with discrepancies resolved by a third author. Data from included studies were abstracted by a single author (SAA) and verified by a second author (AAA). Data were extracted in a pre-piloted data extraction tool developed in Airtable (https://airtable.com)—a commercially available web-based relational database tool. We extracted key characteristics of each study, including (1) study location; (2) methods: study design, dates and duration of study and follow-up, and number and type of sites; (3) study population: number, age, sex, and inclusion/exclusion criteria; (4) intervention and comparator details; (5) outcomes: ART uptake, retention in care, viral suppression, mortality, adherence, and adverse events, extracted when possible with numerators, denominators, and/or measures of association; and (6) indicators of risk of bias. Any discrepancies were resolved by discussion among the authors. The Cochrane or Newcastle–Ottawa Scale tools were used to assess risk of bias [13,14].

### Assessment of study implementation characteristics

We additionally characterized studies according to PRECIS-2 criteria for how pragmatic or explanatory included studies were—exploring eligibility assessments, recruitment procedures, settings, organizational characteristics, flexibility in intervention delivery and adherence, follow-up, primary outcome reporting, and primary analyses [15]. Although the tool is optimized for randomized control trial (RCT) design, we applied PRECIS-2 concepts across all studies. We also explored reporting of implementation outcomes in primary and additional study publications across 8 domains: acceptability, adoption, appropriateness, implementation cost, feasibility, fidelity, adaptation, penetration, and sustainability [16].

### Data synthesis and statistical analysis

For pairwise meta-analyses, we used random effects generic inverse variance meta-analytic models; we evaluated ART uptake (initiation) among all HIV-positive individuals, and for retention in care and viral suppression we assessed these outcomes among all who initiated ART. We determined risk ratios (RRs) and 95% confidence intervals for all outcomes and additionally present risk differences (RDs) where absolute effects were deemed valuable for interpretation. For cluster randomized trials we calculated the design effect using methods outlined in the Cochrane handbook (where dichotomous counts from each study are divided by the quantity 1 + [*M −* 1] × ICC, where *M* is the average cluster size and ICC is the intra-cluster correlation coefficient) to adjust estimates if we could not incorporate adjusted estimates directly from study publications. We used Mantel–Haenszel methods to generate pooled estimates of binary data. We evaluated heterogeneity qualitatively through examining forest plots and quantitatively through examination of the *I*^2^ statistic. Between-study variance was evaluated using the Paule–Mandel estimator for Tau2 and the associated *I*^2^ statistic [17]. We used subgroup analyses to explore heterogeneity. For each outcome, we generated forest plots overall and subgrouped where relevant by study design, population type, and implementation features. We conducted tests for subgroup differences to determine if cohort and RCT data could be pooled. R statistical software was used for all analyses [18].

### Evidence appraisal

We evaluated the certainty/quality of the body of evidence contributing to the pooled effect estimate for each outcome using criteria recommended by the GRADE Working Group, including risk of bias, inconsistency, indirectness, imprecision, and other domains [19–22].

## Results

**Fig 1:**
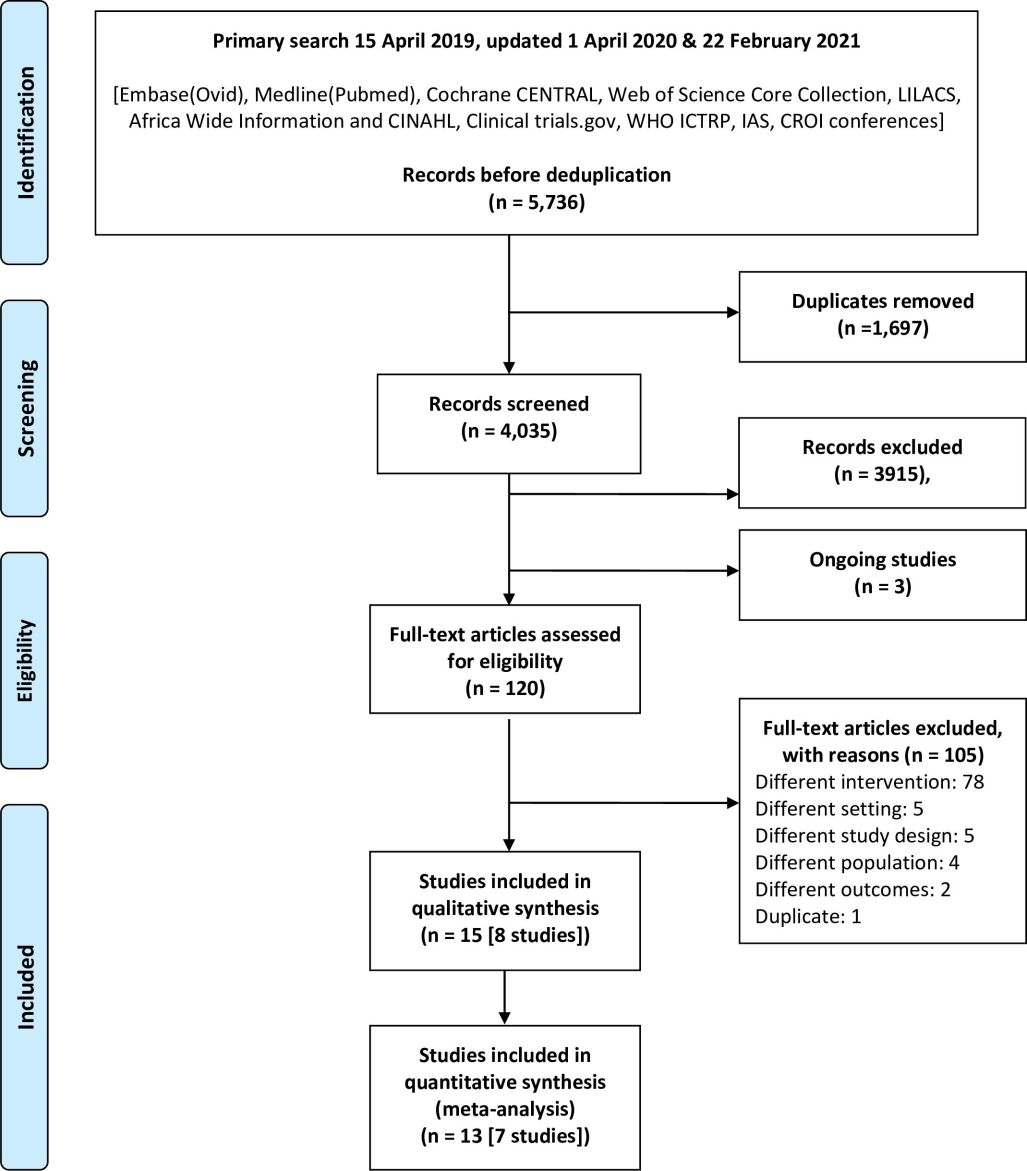
PRISMA flow diagram. CROI, Conference on Retroviruses and Opportunistic Infections; IAS, International AIDS Society; ICTRP, International Clinical Trials Registry Platform.

Searches were conducted between 1 January 2013 and 22 February 2021 and yielded 4,035 abstracts for screening after deduplication. We identified 120 records for full-text screening and 3 possibly eligible ongoing studies; 105 studies were excluded with reasons, and 15 publications representing 8 studies were included in the qualitative synthesis, with 7 studies included in meta-analyses (Fig 1).

Four studies were RCTs, 2 individually randomized [23–25] and 2 cluster randomized [26,27]; 3 were cohort studies with a comparison arm [28–30], and 1 study was a single-arm cohort study [31]. Two studies were conducted in Nigeria [29,31] and Lesotho [23,27] each, and 1 each was conducted in South Africa [25], Uganda [32], Malawi [26], Tanzania [28], and Haiti [30]. Five studies were conducted in general populations, 2 included key population groups, and 1 included adolescents (Table 1).

**Table 1:** General description of included studies.

Study	Study Design	Country	Setting	Number of HIV+ participants	Population type	Eligibility criteria for inclusion
Barnabas 2020 [24,25]	RCT	South Africa and Uganda	Rural regions	1,315	General HIV endemic communities	ART naïve, WHO stage 1–3, CD4 cell count > 100/μL, not pregnant or breastfeeding, negative TB symptom screen, normal renal function
Ibiloye 2018 [31]	Cohort (single arm)	Nigeria	Three districts in a central state	935	Several key populations: FSWs, MSM, PWID	Any CD4 cell count
Labhardt 2018 [23,33,35]	RCT	Lesotho	Rural northern region	274	General HIV endemic communities	ART naïve, WHO stage 1–3, not pregnant or breastfeeding, no chronic illness, CRAG negative
Amstutz 2021 [27]	RCT (cluster)	Lesotho	Rural northern region	257	General HIV endemic community	ART naïve; weight > 35 kg; no other chronic condition; physical, mental, and emotional ability to participate; remaining in district for HIV care
MacPherson 2014 [26]	RCT (cluster)	Malawi	Blantyre (urban center)	768	General HIV endemic communities	CD4 cell count < 350/μL or WHO stage 3 or 4 or pregnant or breastfeeding
Oladele 2018 [29]*	Cohort	Nigeria	Fourteen donor-funded high-HIV-burden districts (urban and rural)	6,270	General HIV endemic communities	CD4 cell count < 500/μL or WHO stage 3 or 4
Reif 2017 [30]**	Cohort	Haiti	Port Au Prince (urban center)	760	Adolescents and young adults	Community care group: any CD4 count; historical cohort: CD4 cell count < 350/μL
Tun 2019 [28,36]	Cohort	Tanzania	High HIV prevalence (major trucking routes)	617	FSWs	Any CD4 count

CRAG, cryptococcal antigen screening; FSW, female sex worker; MSM, men who have sex with men; PWID, people who inject drugs; RCT, randomized controlled trial; TB, tuberculosis.

*Data restricted to patients testing for HIV in the community in the post-intervention period for intervention and control areas.

**Unpublished data from conference abstract only.

Studies incorporated several differentiated service delivery features including task shifting/sharing and changes in the location and frequency of services (Table 2). Community ART initiation was in most cases delivered by a small team including a nurse and counselor or a village health worker, but in some studies involved a larger team, including lab technicians, pharmacists, doctors, and additional community lay workers [28,29,31]. Within the community setting, 2 studies initiated ART in the home, and the remaining studies initiated ART at community venues (such as mobile vans, individual homes, and other community venues). All studies paired community ART initiation with community-based HIV testing strategies. Two studies included HIV self-testing strategies; in one this was the primary method of HIV testing [26], and in another HIV self-tests were distributed to those who declined testing and those away from home in a subset of participants [27]. The location of ART refill collection (after ART initiation) varied, with half of the studies having participants collect ART refills in the community [28,30,31] and the other half having participants collect ART refills at the health facility [23,26,29]; Once stabilized on ART, those receiving facility-based ART refills received ART refills every 3 months. All studies initiated ART rapidly, either on the same day as testing (5 studies) or within 7 days (3 studies). Six comparative studies compared community ART initiation to facility-based ART initiation, with a similar subsequent frequency of ART refills in all except 1 study [23], where refill frequency was monthly in the facility arm compared to every 3 months in the community arm. One comparative study compared different ART refill strategies across 2 community ART arms [27]. In several studies, community ART initiation strategies were also combined with additional demand creation [26,31], enhanced support strategies [28–30], or SMS reminders [27]—beyond what was offered in the facility-based initiation (standard of care) arm.

**Table 2:** Intervention strategy details.

Study	Community ART group								Comparator groups
	Community ART initiation team	HIV testing site	ART initiation site	Time to ART start	Immediate follow-up	ART refill frequency	ART refill location	Additional support/interventions beyond routine care	
Barnabas 2020 [24,25]	Nurse, lay provider, ± driver	Community	Mobile van	≤7 days	Phone call at 7 days, in person at 1 month	Every 3 months	Mobile van	Food parcels provided at each study visit, quarterly phone calls	Two comparisons: (1) facility ART initiation with facility ART maintenance, (2) facility ART initiation and community ART maintenance
Ibiloye 2018 [31]	Community facilitator, ART clinician, nurse, counselor, pharmacist, lab technician	Community	Choice of outreach venues (CBO offices, hotels/guest houses)	Same day	Not described	Not described	Outreach venues in community	STI care	No comparator
Labhardt 2018 [23,33,35]	Nurse, counselor	Home	Home	Same day	12–16 days and 6 weeks at health facility	Every 3 months	Health facility	Medical care at health facility	Facility ART initiation and maintenance, monthly clinic visits
Amstutz 2021 [27]	Nurse, counselor	Home	Home	Same day	12–16 days at VHW’s home	Every 3 months	VHW’s home	Monthly ART reminder via SMS, viral load result triggered SMS	Home-based ART initiation and facility ART follow-up and maintenance
MacPherson 2014 [26]	Nurse, counselor	Home (HIV self-test)	Home	≤7 days	2–4 weeks at health facility	Every 3 months	Health facility	Demand creation (HIV self-test and home ART awareness campaigns)	Facility ART initiation and maintenance
Oladele 2018 [29]	Doctor, counselor, pharmacist, lab technician, nurse, community lay workers	Community	Point of identification in community	Same day	Phone calls/SMS/home visit every 3 days for 2 weeks, first facility refill at 1 month	Every 3 months	Health facility	Community mobilization campaigns, task sharing between providers and lay counselors	Facility ART initiation and maintenance
Reif 2017 [30,37]	Nurse, peer educator	Facility and community	Community center	Same day	At 1 month	Monthly	Community center	Integrated clinical care, FP, STI care, peer support	Facility ART initiation and maintenance, monthly clinic visits, routine facility-based support groups
Tun 2019 [28,36]	Clinician, nurse, lab technician, peer educator	Community	Mobile tent, home	≤7 days	Not described	Not described	Mobile tent, home	STI care, condom distribution, FP, IPV care, TB screening, CaCx screening, escorted referrals	Facility ART initiation and maintenance

CBO, community-based organization; CaCx, cervical cancer; FP, family planning; IPV, intimate partner violence; STI, sexually transmitted infection; TB, tuberculosis; VHW, village health worker.

Macpherson 2014: 36% of those offered home ART in the home group chose facility ART initiation (64% selected home art initiation); Amstutz 2021: 6- and 12-month ART refill visits were at health facility due to viral load measurement.

Data from RCTs were generally judged as having high methodological quality (low risk of bias), and observational data as having poor quality, as assessed by risk of bias tools (Table 3; S2 Appendix). Observational studies had several methodological limitations, primarily related to lack of comparability of study arms and inclusion of data that were not adjusted for baseline imbalances in the pairwise meta-analysis.

**Table 3:** Risk of bias assessments.

Study	ART uptake/initiation among HIV+ individuals	Retention in care among ART initiators	Viral suppression among ART initiators	Adherence among ART initiators	Mortality among HIV+ individuals
Barnabas 2020	NA	NA	Low risk	NA	NA
Labhardt 2018	Low risk	Low risk	Low risk	NA	Some concerns
Amstutz 2021^1^	NA	Some concerns	Some concerns	NA	Some concerns
MacPherson 2014	Low risk	Low risk	NA	High risk	Some concerns
Oladele 2018^2^	Poor quality	NA	NA	NA	NA
Reif 2017^3^	Poor quality	Poor quality	NA	NA	NA
Tun 2019^4^	Poor quality	Poor quality	Poor quality	Poor quality	Poor quality

NA, not applicable.

Assessments based on Cochrane RoB 1 tool for randomized controlled trials (high risk, some concerns, low risk) or Newcastle–Ottawa Scale for cohort studies (poor quality, good quality). Detailed assessments available in S2 Appendix.

^1^Allocation concealment was not possible; recruiting teams were aware of household assignments prior to recruitment; participants were, however, unaware of assignment during recruitment.

^2^Comparison group (facility referral for ART initiation) very small compared to home ART group; fundamental differences between community and facility art initiation groups (facility groups urban and with lower HIV prevalence); unadjusted numbers used in this analysis.

^3^Overall risk of bias influenced by different sources of comparison (historical cohort with CD4 cell count < 350/μL) and intervention group; unadjusted estimates used in analysis without controlling for any baseline characteristics; in addition, the comparative analysis remains unpublished.

^4^Comparison group drawn from a different region; ascertainment of exposure not described; unadjusted estimates used in analysis; baseline imbalance in the group characteristics; intervention group had substantially more newly diagnosed participants, which could affect uptake and retention.

We used PRECIS-2 criteria to assess how pragmatic or explanatory included studies were (Tables 4 and S2): Overall, studies were highly pragmatic—conducted in real-world settings, with flexible approaches to intervention delivery and few additional measures to ensure adherence to ART beyond what would occur in routine practice. RCTs were on average less pragmatic than cohort studies. Study procedures in trials that appeared less applicable in the “real world” setting included the following: (1) more restrictive inclusion criteria that included, for example, CD4 count measurement, or exclusion of those who were pregnant, breastfeeding, or had chronic conditions [25,27,34]; (2) expertise and resources used to deliver the intervention, with large, well-trained, multidisciplinary teams initiating ART in the community in some studies [23,26], or the provision of food parcels at visits [25]; and (3) extensive patient follow-up, where tracing efforts appeared more rigorous than what may occur in routine practice [23]—approaches that may not be entirely reproducible at scale.

**Table 4:** PRECIS-2 criteria/score.

Study	Eligibility	Recruitment	Setting	Organization	Flexibility: Delivery	Flexibility: Adherence	Follow-up	Primary outcome	Primary analysis
	Who is selected to participate in the trial?	How are participants recruited into the trial?	Where is the trial being done?	What expertise and resources are needed to deliver the intervention?	How should the intervention be delivered?	What measures are in place to make sure participants adhere to the intervention?	How closely are participants followed up?	How relevant is it to participants?	To what extent are all data included?
Barnabas 2020	4	4	5	3	4	5	5	5	5
Ibiloye 2018	5	4	5	3	5	5	3	5	5
Labhardt 2018	4	3	5	3	4	4	2	5	5
Amstutz 2021	5	4	5	3	4	5	4	5	5
MacPherson 2014	5	4	4	3	3	5	4	5	5
Oladele 2018	5	5	3	3	5	5	5	5	4
Reif 2017	5	5	4	4	5	5	4	5	4
Tun 2019	5	3	5	4	4	5	3	5	5

Value of 5 (dark green) represents a very pragmatic approach and a value of 1 (yellow) represents a very explanatory approach. Detailed assessments presented in S2 Table.

### ART initiation

**Fig 2:**
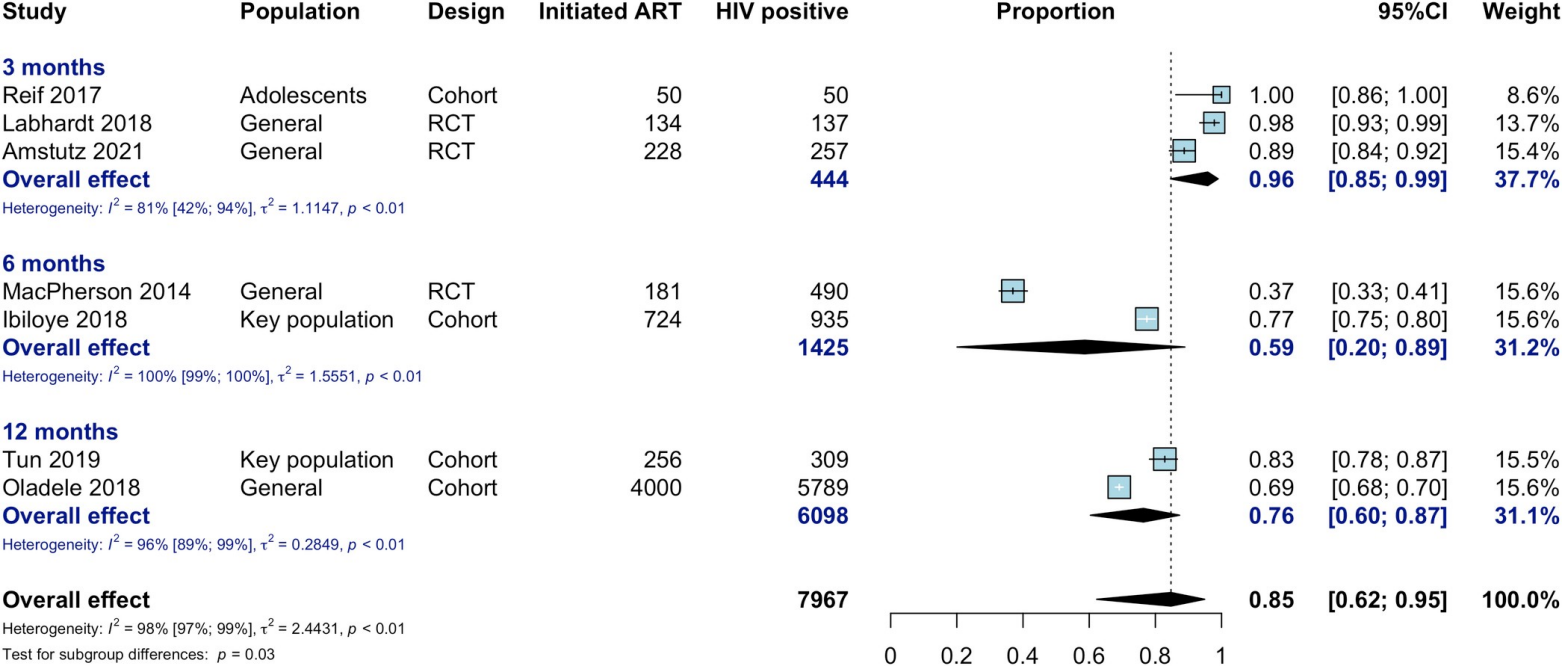
ART uptake in community ART initiation study arms, among HIV-positive and by outcome measurement time point. Tun 2019 was conducted in female sex workers; Ibiloye 2018 included female sex workers, men who have sex with men, people who inject drugs, and partners of individuals in these key populations.

Seven studies reported on ART initiation after offer of community ART; ART uptake among those testing HIV-positive was high overall (85%), but there was substantial heterogeneity of measurement time points, and uptake across studies ranged from 37% to 100% (Fig 2). The lowest ART uptake (37%) was seen in a study where HIV self-testing was conducted and paired with community ART initiation [26]. A single-arm study conducted in key populations had an overall uptake of 77%, but this varied across key population subgroups, with uptake of 75% in female sex workers (FSWs), followed by men who have sex with men (68%) and people who inject drugs (PWID) (53%); partners of key populations in this study had generally high community ART uptake (93%) [31].

**Fig 3:**
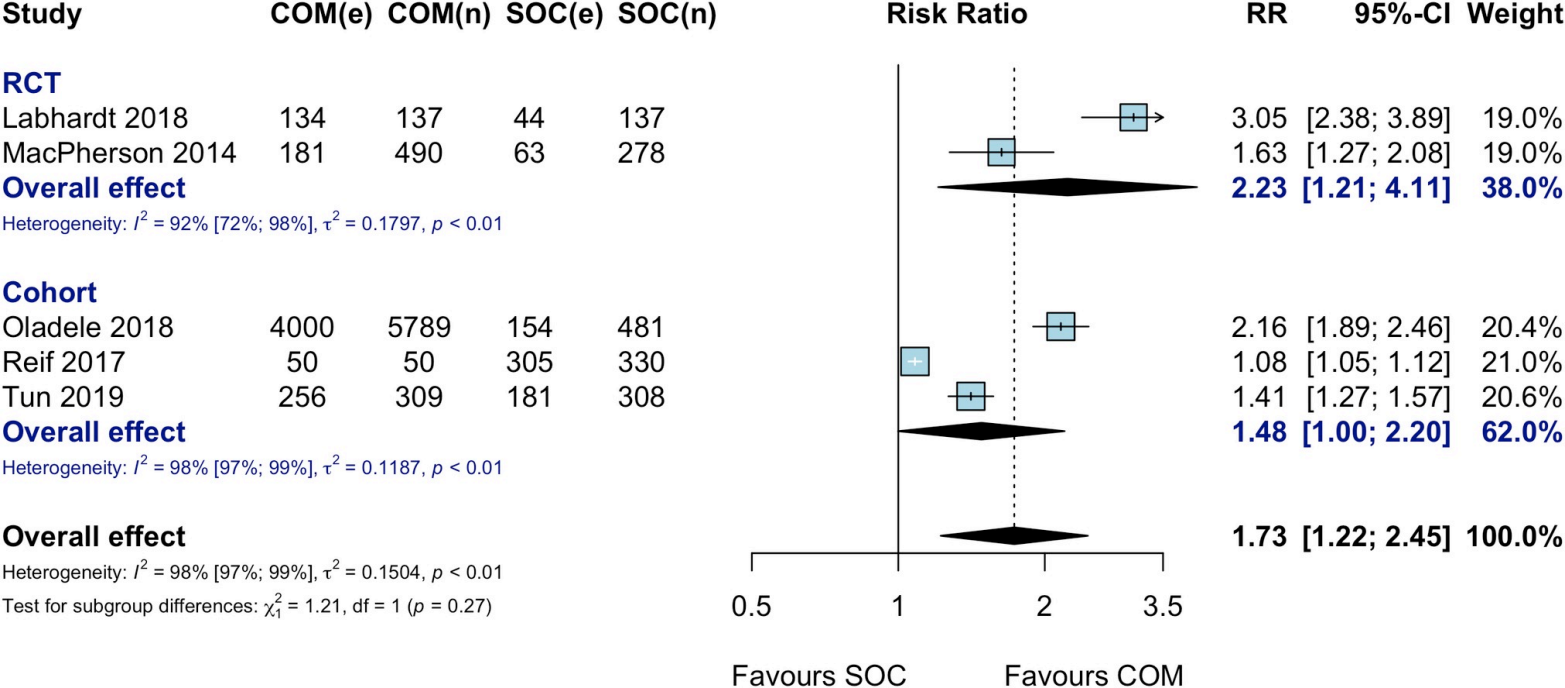
ART uptake: Community ART initiation versus standard of care among HIV-positive individuals, by study design. COM, community ART initiation; e, number of events; n, number of participants; RCT, randomized controlled trial; RR, risk ratio; SOC, standard of care. MacPherson 2014 cluster-adjusted effect estimate based on the Cochrane method of adjusting for the design effect.

Five studies compared community ART initiation to facility-based ART initiation and refill (standard of care). The meta-analysis of these studies showed higher ART initiation when ART was offered in the community (Fig 3; RR 1.73, 95% CI 1.22 to 2.45; *I*^2^ 98%), which translated to an absolute risk difference of 30% (95% CI 10% to 50%) (S1 Fig).

When subgrouped by study design, a stronger effect was seen in RCTs (RR 2.23, 95% CI 1.21 to 4.11) as compared to cohort studies (RR 1.48, 95% CI 1.00 to 2.20); this difference accounted for some of the heterogeneity seen in the overall analysis but not all. There was greater heterogeneity of effect estimates among cohort studies compared to RCTs: 2 cohort studies, one conducted in FSWs (RR 1.41, 95% CI 1.27 to 1.57) and another in adolescents (RR 1.08, 95% CI 1.05 to 1.12), showed smaller differences in ART uptake compared to studies conducted in general HIV endemic communities (RR 2.20, 95% CI 1.56 to 3.12) (S2 Fig). Home ART initiation (explored only in RCTs) had comparable ART uptake (RR 2.23, 95% CI 1.21 to 4.11) to ART offered at other venues in the community (RR 1.48, 95% CI 1.00 to 2.20) (S3 Fig). Same-day ART initiation (RR 1.91, 95% CI 1.05 to 3.46) showed similar ART uptake to initiation within 7 days (RR 1.45, 95% CI 1.30 to 1.62) (S4 Fig).

### Retention in care

**Fig 4:**
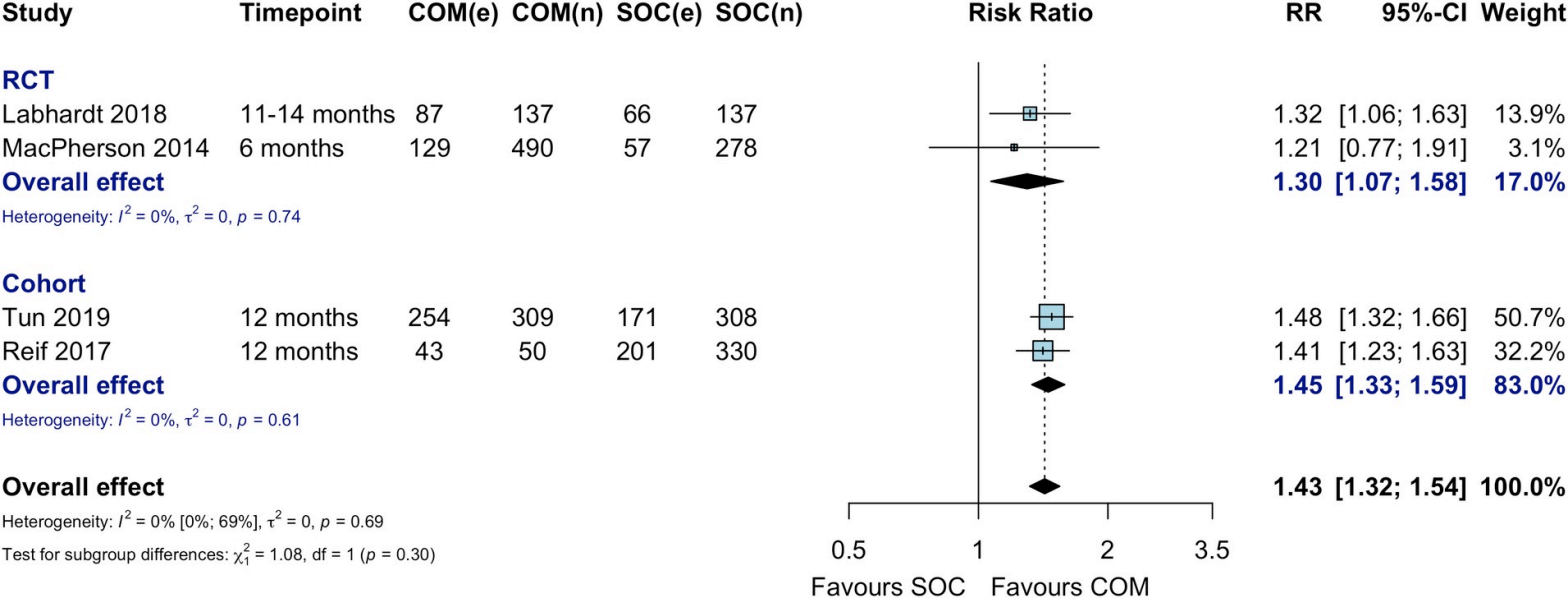
ART retention at 6–12 months among HIV-positive individuals. COM, community ART initiation; e, number of events; n, number of participants; RCT, randomized controlled trial; RR, risk ratio; SOC, standard of care. MacPherson 2014 reflects cluster-adjusted numbers based on the Cochrane method of adjusting for the design effect.

Four studies compared retention in care between community ART initiation and facility ART initiation and maintenance (standard of care) among HIV-positive individuals at 6–12 months [23,26,28,30]. Retention was higher in the community ART initiation group compared to the facility ART initiation group (RR 1.43, 95% CI 1.32 to 1.54), which translated to a risk difference of 19% (95% CI 11% to 28%) (Figs 4 and S5).

**Fig 5:**
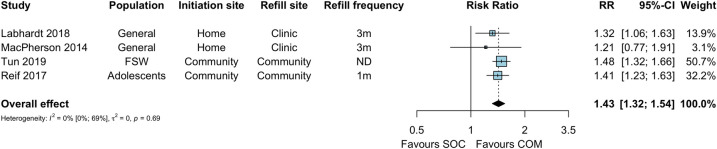
ART retention at 6–12 months among HIV-positive individuals, by implementation strategy. COM, community ART initiation; FSW, female sex workers; ND, not determined; RR, risk ratio; SOC, standard of care. MacPherson 2014 reflects cluster-adjusted numbers based on the Cochrane method of adjusting for the design effect.

When compared to standard of care (facility-based ART initiation and refills), retention did not appear to differ by population type, ART initiation site, refill site, or frequency of ART refill (Fig 5). One study additionally reported retention among HIV-positive individuals at 24 months and found no difference between community ART initiation and facility ART initiation and maintenance (RD 5%, 95% CI −16% to 16%, *p* = 0.380) at this time point [33]. In pooled analyses restricted to those who initiated ART, retention was no different between study arms (S6 Fig; RR 1.15, 95% CI 0.99 to 1.33). The single-arm study Ibiloye 2018 [31] reported 73.2% retention in care among key populations of individuals who initiated ART in the community ART arm after 7 months of follow-up.

An additional study that compared 2 community ART initiation arms reported slightly better retention at 12 months when community ART initiation was combined with facility refills (71%) compared to community-based ART refills (60%); this however did not reach statistical significance (RD −12%, 95% CI −23% to 0.3%) [27].

### Viral suppression

**Fig 6:**
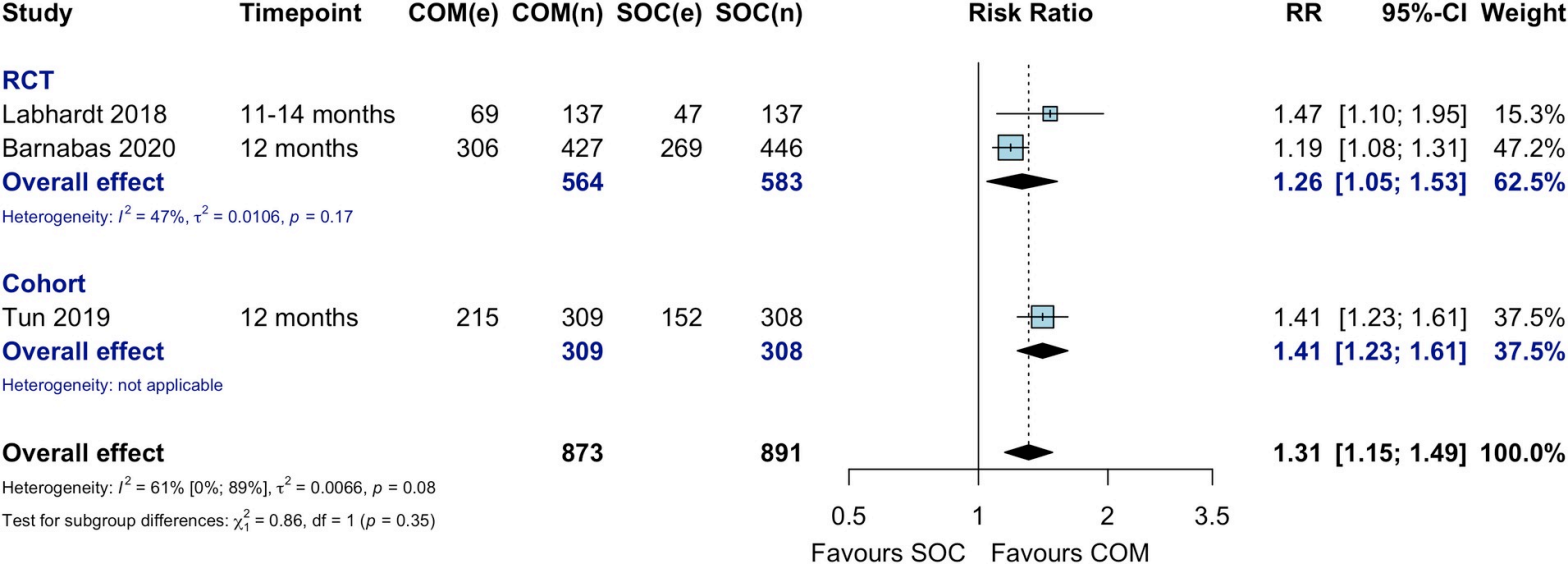
Viral suppression among HIV-positive individuals at 12 months: Community ART initiation versus facility-based care. COM, community ART initiation; e, number of events; n, number of participants; RCT, randomized controlled trial; RR, risk ratio; SOC, standard of care.

Three studies compared viral suppression between community ART initiation and facility ART initiation and maintenance (standard of care) arms among HIV-positive individuals at 12 months; this comparison showed better viral suppression in the community ART group (RR 1.31, 95% CI 1.15 to 1.49) (Fig 6). One study additionally reported 24-month outcomes, which showed no difference in viral load suppression in the community ART arm compared to the facility ART arm (RD 3%, 95% CI −9% to 15%, *p* = 0.28) at this time point [33]. The threshold for viral load suppression ranged from less than 100 copies/mL [23], to 1,000 copies/mL [28]. In one study, viral suppression in the community ART arm was higher when data were restricted to men from a South African subgroup (RR 1.39, 95% CI 1.17 to 1.66) [25]. Meta-analysis of viral suppression restricted to those who initiated ART showed no difference in viral suppression between treatment arms at 12 months (RR 1.05, 95% CI 0.86 to 1.29) (S7 Fig).

Two studies compared community ART initiation with hybrid community–facility ART strategies, including either facility-based ART initiation or facility-based ART refill (Table 5). One compared community ART initiation and refills versus facility ART initiation with community ART refills [24,25] and found no difference in viral suppression between these 2 strategies. When analysis was restricted to the South African male subgroup, community ART initiation and maintenance appeared to have better viral suppression than if ART was initiated at the facility and maintained in the community (RR 1.26, 95% CI 1.04 to 1.51) in this study. Another study compared 2 community ART initiation strategies, one with ART refills delivered in the community versus another with refills collected at the health facility; this comparison showed no difference in viral suppression at 12 months between refill strategy arms (RD −7%, 95% CI −20% to 6%) [27].

**Table 5:** Viral suppression among HIV-positive individuals at 12 months: Community ART initiation and refill versus community–facility hybrid initiation and refill strategies.

Study	Community ART		Hybrid community–facility ART		Effect estimate, 95% CI
	ART initiation	ART refill site	ART initiation	ART refill site	
Barnabas 2020	Community	Community	Facility	Community	RR 1.08, 95% CI 0.98 to 1.19
Amstutz 2021	Community	Community	Community	Facility	RD −7%, 95% CI −20% to 6%

RD, risk difference; RR, risk ratio.

### ART adherence

**Fig 7:**
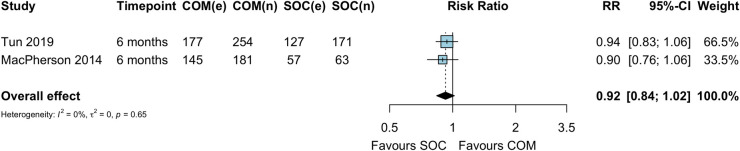
ART adherence among ART initiators at 6 months. COM, community ART initiation; e, number of events; n, number of participants; RR, risk ratio; SOC, standard of care. MacPherson 2014 reflects cluster-adjusted estimates based on the Cochrane method of adjusting for the design effect.

There was no difference in ART adherence among ART initiators at 6 months in the 2 studies contributing to this comparative analysis (RR 0.92, 95% CI 0.84 to 1.02) (Fig 7). Adherence was assessed as not missing a single dose in the past 4 days [26] or not missing a dose in the past 7 days as assessed by self-report [28].

### Mortality

**Fig 8:**
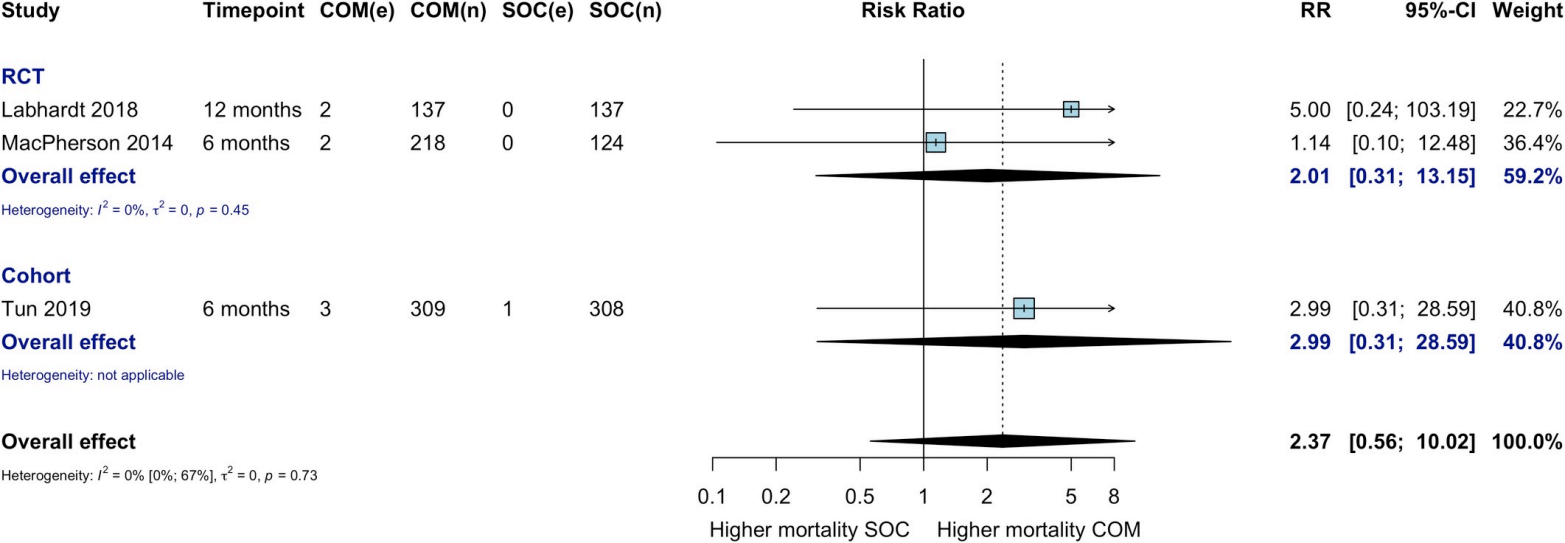
Mortality among HIV-positive individuals at 12 months. COM, community ART initiation; e, number of events; n, number of participants; RCT, randomized controlled trial; RR, risk ratio; SOC, standard of care.

Overall, there were few events contributing to this outcome. Three studies contributed to the comparative analysis of mortality (at 6 to 12 months), showing no difference in mortality among those who initiated ART in the community compared with those who initiated in the health facility (Fig 8; RR 2.37, 95% CI 0.56 to 10.05). The Ibiloye 2018 study reported overall mortality in its community ART non-comparative cohort study at 3, 6, and 9 months on ART as 3.4%, 3.7%, and 3.9%, respectively. Additionally, Amstutz et al. reported 5% (6/118) mortality in the community ART initiation and refill arm, compared to 0% (0/139) mortality in the hybrid community ART initiation and facility refill arm [27].

### Adverse events and social harms

There was variable adverse event reporting, including mild, serious, and severe adverse events, social harms, and opportunistic infection incidence (Table 6). Severe adverse events in community ART initiation arms ranged from 1% to 6% and in facility ART initiation arms ranged from 1% to 2% [23,25,30,33]. There were very few opportunistic infections or social harms; this was however infrequently reported in the included studies.

**Table 6:** Adverse events among HIV-positive.

Study	Adverse events		Opportunistic infections		Social harms	
	Community ART initiation	Facility ART initiation	Community ART initiation	Facility ART initiation	Community ART initiation	Facility ART initiation
Labhardt 2018	6 (4%) events—2 rash, 1 nausea, 1 dizziness, 1 gynecomastia, 1 elevated alanine aminotransferase level	2 events (1%)—2 rash	0 TB cases	2 (1%) TB cases	—	—
Reif 2017	—	—	—	—	0—increased stigma or unintended disclosure	0—increased stigma or unintended disclosure
Barnabas 2020 (a)	7 (1%) severe and 7 (1%) serious adverse events	8 (2%) severe and 2 (0.4%) serious adverse events	—	—	2 events	0 events
Barnabas 2020 (b)	—	5 (1%) severe and 4 (1%) serious adverse events	—	—	—	0 events
Amstutz 2021 (a)	7 (6%) events—serious adverse events	—	—	—	—	—
Amstutz 2021 (b)	7 (3%) events—serious adverse events	—	—	—	—	—

Barnabas 2020 (a): community ART initiation arm combined with community ART refills. Barnabas 2020 (b): community ART initiation arm combined with facility ART refills. Amstutz 2021 (a): community ART initiation combined with community ART refills. Amstutz 2021 (b): community ART initiation combined with facility ART refills.

### Certainty of review findings (GRADE assessment)

The certainty of the evidence (a combined assessment of strength of association, methodological quality, heterogeneity, and external validity) for the pooled (RCT and cohort) data on primary outcomes of uptake, retention, and viral suppression among HIV-positive individuals was graded as low to moderate (Table 7); effect estimates were downgraded due to high risk of bias in the contributing observational studies (Table 7). Pooled estimates for adherence were similarly graded as low certainty evidence, due to the inclusion of self-reported outcomes and poor methodological quality. Very few events contributed to the mortality analysis, resulting in very low certainty evidence for this outcome.

**Table 7:** Review evidence certainty assessment (GRADE): Community ART initiation versus facility ART initiation.

Certainty assessment							Number of patients		Effect estimate (95% CI)	Certainty
Number of studies	Study design	Risk of bias	Inconsistency	Indirectness	Imprecision	Other	Community ART initiation	Facility ART initiation		
**ART initiation among PLWH**										
5	RCTs and observational	Serious^a^	Serious^b^	Not serious	Not serious	None	4,621/6,466 (71.5%)	747/1,534 (48.7%)	RR 1.73 (1.22 to 2.45)	⨁⨁◯◯ LOW
2	RCTs	Not serious	Serious^b^	Not serious	Serious^c^	None	315/503 (62.6%)	107/415 (25.8%)	RR 2.23 (1.21 to 4.11)	⨁⨁◯◯ LOW
3	Observational	Serious^a^	Serious^b^	Not serious	Not serious	None	4,306/6,148 (70.0%)	640/1,119 (57.2%)	RR 1.48 (1.00 to 2.20)	⨁◯◯◯ VERY LOW
**Retention in care among PLWH at 6–12 months**										
4	RCTs and observational	Serious^a^	Not serious	Not serious	Not serious	None	513/716 (71.6%)	459/993 (46.2%)	RR 1.44 (1.33 to 1.56)	⨁⨁⨁◯ MODERATE
2	RCTs	Not serious	Not serious	Not serious	Serious^c^	None	216/627 (34.4%)	123/415 (29.6%)	RR 1.30 (1.07 to 1.58)	⨁⨁⨁◯ MODERATE
2	Observational	Serious^a^	Not serious	Not serious	Not serious	None	297/359 (82.7%)	372/638 (58.3%)	RR 1.45 (1.33 to 1.59)	⨁⨁◯◯ LOW
**Retention in care among PLWH at 24 months**										
1	RCT	Not serious	Not serious	Not serious	Very serious^c^	None	88/137 (64%)	81/137(59%)	RD 5% (−16% to 16%)	⨁⨁⨁◯ LOW
**Viral suppression among PLWH at 12 months**										
3	RCTs and observational	Serious^a^	Not serious	Not serious	Not serious	None	590/873 (67.6%)	468/891 (52.5%)	RR 1.31 (1.15 to 1.49)	⨁⨁⨁◯ MODERATE
2	RCTs	Not serious	Not serious	Not serious	Not serious	None	375/564 (66.5%)	316/583 (54.2%)	RR 1.26 (1.05 to 1.53)	⨁⨁⨁⨁ HIGH
1	Observational	Serious^a^	Not serious	Not serious	Not serious	None	215/309 (69.6%)	152/308 (49.4%)	RR 1.41 (1.23 to 1.61)	⨁◯◯◯ VERY LOW
**Viral suppression among PLWH at 24 months**										
1	RCT	Not serious	Not serious	Not serious	Very serious^c^	None	78/137 (56.9%)	74/137 (54.0%)	RD 3% (−9% to 15%)	⨁◯◯◯ VERY LOW
**Adherence among ART initiates at 6 months**										
2	RCTs and observational	Serious^a^	Not serious	Not serious	Serious^c^	None	322/378 (85.2%)	184/212 (86.8%)	RR 0.92 (0.84 to 1.02)	⨁⨁◯◯ LOW
**Mortality among PLWH at 6–12 months**										
3	RCTs and observational	Serious^a^	Not serious	Not serious	Very serious^c^	None	7/664 (1.1%)	1/572 (0.1%)	RR 2.37 (0.56 to 10.02)	⨁◯◯◯ VERY LOW

PLWH, people living with HIV; RCT, randomized cont rolled trial; RD, risk difference; RR, risk ratio.

^a^Included observational studies that have methodological concerns—comparison groups drawn from different populations and baseline imbalances were not accounted for in the analysis.

^b^I^2^ statistic >90%

^c^Few studies and events

### Implementation outcomes

Few studies reported on implementation outcomes of community ART initiation, 2 studies reported on cost, and no studies reported on acceptability, penetration, adoption, fidelity, adaptations, feasibility, or sustainability related to community ART initiation.

Of the 2 studies reporting on costs, one reported on the community ART initiation arm only [26], and the second reported cost comparisons of facility ART initiation and community ART initiation across 3 study settings [25]. The Barnabas 2020 study demonstrated some variability across settings, with the cost per person virally suppressed higher with community-based ART initiation compared to facility ART initiation in 2 of the settings (Table 8).

**Table 8:** Cost analyses reported in included studies.

Study	Currency	Total cost components	Cost measures reported	Facility ART initiation	Community ART initiation
MacPherson 2014 (Malawi)	2012 US dollars	Community ART initiation (60.3%), staff training (0.6%), community sensitization (0.5%), drugs (3%), consumables (13.8%), equipment (8.1%), other recurrent items (13.7%); excludes HIV testing costs	Average cost per participant assessed	—	$97
			Average cost per participant initiated on ART	—	$127
Barnabas 2020 (South Africa^a^)	2018 US dollars	Cost of ART, trimethoprim-sulfamethoxazole, laboratory testing, personnel, supplies, fuel, and overheads	Annual cost of community-based ART per client	$249	$312
			Annual cost per person virally suppressed	$422	$452
Barnabas 2020 (South Africa^b^)	2018 US dollars	Cost of ART, trimethoprim-sulfamethoxazole, laboratory testing, personnel, supplies, fuel, and overheads	Annual cost of community-based ART per client	$249	$308
			Annual cost per person virally suppressed	$402	$380
Barnabas 2020 (Uganda)	2018 US dollars	Cost of ART, trimethoprim-sulfamethoxazole, laboratory testing, personnel, supplies, fuel, and overheads	Annual cost of community-based ART per client	$163	$217
			Annual cost per person virally suppressed	$214	$275

^a^Midlands Kwazulu-Natal, South Africa.

^b^Northern Kwazulu-Natal, South Africa.

### Ongoing studies

We identified 3 ongoing studies being conducted in Indonesia [38], Zimbabwe [39], and Puerto Rico [40]. One study includes adolescents, and 2 studies include key population groups (S3 Table).

## Discussion

In this systematic review, we found that making ART initiation available in the community led to increases in ART uptake, better retention, and improved viral suppression (over the course of 1 year after ART offer), compared to traditional facility-based ART initiation. Models offering ART initiation in the community increased uptake by 30% and 1-year retention by 19% compared to initiation and maintenance at a traditional health facility. This finding of higher ART uptake in the community ART arm was consistent across study designs and various implementation methods. In one head-to-head comparison of alternative ART refill distribution strategies after community ART initiation, 1-year retention and viral suppression was comparable between community ART refill and facility refill. Another study measured 2-year outcomes and found viral suppression and retention to be no different at this time point between those offered ART initiation in the community and those offered facility initiation. There were too few events to confidently determine the effect of community ART initiation on adherence and mortality. Similarly, few studies reported on adverse events or social harms. These findings were based on the synthesis of 8 studies conducted primarily in low- and middle-income African countries with high to moderate HIV burden, representing diversity in geographical locations and population groups in these settings.

The effect of community-based models for initiating ART had consistent effects across implementation strategies and population subgroups. Community ART was provided through a variety of distribution models: ART was provided at home, in mobile vans and tents, or at community locations; ART was initiated by large multidisciplinary teams in some studies, and in others was primarily nurse driven; and ART refills were subsequently distributed either in the community or at health facilities. In comparative analyses, community ART initiation was explored in general HIV endemic communities, men, adolescents, and FSWs, and in one study was seen to be particularly beneficial for men—suggesting that this may be an additional strategy for improving engagement in HIV services for men, who do not routinely attend health services [8,41]. Among key populations, ART uptake was modest, with the lowest uptake reported in PWID; however, comparative analyses also showed improved treatment outcomes for these groups compared to facility-based offer of ART. Future studies focused on population subgroups could help clarify which implementation strategies are most effective, in which settings, and for whom.

Community ART initiation has the potential to expand differentiated service delivery models and move the entire HIV care cascade into the community, a service delivery approach that both addresses structural barriers to attending health services for people living with HIV (PLWH) and is highly relevant during the COVID-19 pandemic, when decongesting health services has become critical—widespread scale-up, however, needs careful consideration. Although the studies in this review were relatively pragmatic in their design, implementation by external partners, additional resources, technical and logistical assistance, and training provided by research teams may mean that more modest outcomes will be seen when these strategies are incorporated into large-scale public health programs with more limited resources [7]. Lessons can be learned from the implementation of community ART models for “stable” HIV-positive people, which demonstrated effectiveness in trials, but when brought to scale highlighted some of the challenges of bringing services into the community, including difficulties with maintaining the ART supply chains, inadequate resources to support community-based staff, patient concerns regarding HIV-related stigma in the community, and patient preferences for facility-based care in some instances [42–45].

The mechanism by which offering ART in the community improves retention and viral suppression appears to be through greater ART uptake and reduced loss to follow-up prior to ART initiation. Once ART has been initiated, outcomes are similar to those of individuals who initiate ART at a health facility. Analyses of the effect of offering ART to all PLWH on retention and viral suppression compared to analyses that were restricted to only those who initiated ART showed remarkably improved retention and viral suppression at 1 year in the former compared to smaller differences between community and facility arms in the latter. This suggests that expanding the number of PLWH who initiate ART by offering initiation in the community could have a substantial impact on reaching ART coverage goals, if these effects can be reproduced at scale [46].

Few studies included in this review assessed implementation outcomes beyond cost. Additional study findings regarding fidelity to intervention protocols, challenges and required adaptations, explorations of variability in PLWH and provider preferences and acceptability, provider and health system adoption, and sustainability could aid future implementation and should be incorporated into future study design and reporting [47–50].

Our review findings were limited by there being few studies for inclusion, the incorporation of observational data of poor methodological quality, and short-term HIV treatment outcome measures. For systematic reviews evaluating implementation strategies, the inclusion of observational and programmatic data is critical; however, in order to generate robust and relevant synthesized results, high-quality evidence is needed. Assessments of the methodological quality of observational studies included in this review showed observational studies to be of low quality according to risk of bias tools; this was in large part due to the inclusion of data with observed baseline imbalances in participant characteristics between intervention and comparison groups. Although some studies conducted analyses (e.g., interrupted time series) to adjust for selection, these model outputs could not be included in meta-analyses, and therefore raw unadjusted data were pooled with RCT data [29]. Results from observational studies were however consistent with RCT effects, with no differences between study design subgroup estimates, supporting the pooling of these results. The majority of studies reported HIV treatment outcomes at 1 year or less, with the exception of one study, where 2-year viral suppression showed more moderate treatment outcomes as compared to outcomes at 1 year [33]; it is therefore difficult to draw conclusions on the long-term outcomes of offering ART initiation in the community.

Based on data from a limited set of studies, community ART initiation appears to increase ART uptake and as a result shows better viral suppression and retention in care compared to facility ART initiation and refill. Future research should explore which community ART initiation and refill models are most effective for specific populations, evaluate strategies outside of the African context, and report on long-term and implementation outcomes, to facilitate the incorporation of these strategies into HIV programs.

## Supporting information

S1 Appendix PubMed search strategy. (DOCX)

S2 Appendix Detailed risk of bias assessments. (DOCX)

S1 Fig ART uptake among HIV-positive individuals: Risk difference. (PNG)

S2 Fig ART uptake among HIV-positive individuals, by population group. (PNG)

S3 Fig ART uptake among HIV-positive individuals, by community ART initiation location. (PNG)

S4 Fig ART uptake among HIV-positive individuals, by time to ART initiation. (PNG)

S5 Fig Retention in care among HIV-positive individuals: Risk difference. (PNG)

S6 Fig Retention in care among ART initiators. (PNG)

S7 Fig Viral suppression among ART initiators. (PNG)

S1 Table PRISMA checklist. (DOC)

S2 Table Detailed PRECIS-2 rating. (DOCX)

S3 Table Ongoing studies. (DOCX)

## Abbreviations

ART: antiretroviral therapy
FSW: female sex worker
PLWH: people living with HIV
PWID: people who inject drugs
RD: risk difference
RR: risk ratio
RCT: randomized controlled trial
WHO: World Health Organization
