# Supplementary material for: Effects of community-based antiretroviral therapy initiation models on HIV treatment outcomes: A systematic review and meta-analysis
Source: PLoS Med. 2021 May 28;18(5):e1003646. doi: 10.1371/journal.pmed.1003646 (PMC8213195; doi:10.1371/journal.pmed.1003646)
Supplement: S1 Appendix — (DOCX) [file pmed.1003646.s001.docx]

**S1 Appendix:** **Medline (PubMed) search strategy**

**#1** Search ((HIV OR hiv-1 OR hiv-2* OR hiv1 OR hiv2 OR hiv infect* OR human immunodeficiency virus OR human immune deficiency virus OR human immuno-deficiency virus OR human immune-deficiency virus OR ((human immun*) AND (deficiency virus)) OR acquired immunodeficiency syndromes OR acquired immune deficiency syndrome OR acquired immuno-deficiency syndrome OR acquired immune-deficiency syndrome OR ((acquired immun*) AND (deficiency syndrome)) OR HIV/AIDS))

**#2** Search ((HIV infections [MeSH] OR HIV [MeSH]))

**#3** Search (#1 OR #2)

**#4** Search ((Antiretroviral* OR ((anti) AND (retroviral*)) OR ARV* OR ART OR "antiretroviral therapy" OR HAART OR ((highly) AND (active) AND (antiretroviral*) AND (therap*)) OR ((anti) AND (hiv)) OR ((anti) AND (acquired immunodeficiency)) OR ((anti) AND (acquired immuno-deficiency)) OR ((anti) AND (acquired immune-deficiency)) OR ((anti) AND (acquired immun*) AND (deficienc*))))

**#5** Search ((antiretroviral agents [Mesh] OR antiretroviral therapy, highly active [Mesh]))

**#6** Search (#4 OR #5)

**#7** Search (#3 AND #6)

**#8** Search (initiate OR initiating OR initiation OR “early treatment” OR “early therapy” OR “earlier treatment” OR “earlier therapy” OR “start therapy” OR “starting therapy” OR “start treatment” OR “starting treatment” OR “beginning therapy” OR “beginning treatment”)[Title/Abstract]

**#9** Search "Early Medical Intervention"[Mesh]

**#10** Search (link or linking OR linkage OR uptake)[Title/Abstract]

**#11** Search (retention OR retain* OR "lost to follow-up" OR LTFU OR “loss-to-follow-up” OR attrition OR "loss to care" OR "loss to program*" OR default* OR engage* OR disengage* OR “retention in care” OR “lost to retention”)[Title/Abstract]

**#12** Search (loss AND "follow up")[Title/Abstract]

**#13** Search (loss AND followup)[Title/Abstract]

**#14** Search (#8 OR #9 OR #10 OR #11 OR #12 OR #13)

**#15** Search (home-base* OR homebase* OR “home based” OR “home base” OR community-based OR “community based” OR “community health” OR “community program” OR “community programme” OR “community management” OR “door to door” OR “door‐to‐door” OR “home care” OR homecare OR home-care OR “home access” OR in‐home OR “community centre” OR “community center” OR “mobile health unit” OR “mobile health units” OR “mobile outreach”)[Title/Abstract]

**#16** Search ((mobile or movable or moveable or traveling or travelling) AND (unit or units or clinic or clinics or center or centers or centre or centres or facility or facilities or hospital or hospitals))

**#17** Search ((mobile or movable or moveable or traveling or travelling) AND (service or services or health* or medicine or care))

**#18** Search ((medical or health* or mobile or clinic or clinical) AND (bus or buses or van or vans or car or cars or truck or trucks or automobile* or trailer* or wagon* or wheel* or vehicle* or taxi*))

**#19** Search ("Community Health Workers"[Mesh] OR "Volunteers"[Mesh] OR "Allied Health Personnel"[Mesh])

**#20** Search (“community worker” OR “community workers” OR “lay health worker” O “lay health workers” OR “village health worker” OR “village health workers” OR “lay health care worker” OR “lay health care workers” OR “health auxiliary” OR “peer educator” OR “peer educators” OR “peer counsellor” OR “peer counsellors” OR “peer counselor” OR “peer counselors” OR “health extension worker” OR “health extension workers” OR “allied health worker” OR “allied health workers” OR “health promoter” OR “health promoters” OR “health assistance” OR “health assistant” OR “health assistants” OR “voluntary worker” OR “voluntary workers” OR “community volunteer” OR “community volunteers” OR “community level workers”)[Title/Abstract

**#21** Search (#15 OR #16 OR #17 OR #18 OR #19 OR #20)

**#22** Search (#21 AND #14 AND #7)

**#23** Search (#22 AND Filters: Publication date from 2013/01/01)
