# Supplementary material for: Effects of community-based antiretroviral therapy initiation models on HIV treatment outcomes: A systematic review and meta-analysis
Source: PLoS Med. 2021 May 28;18(5):e1003646. doi: 10.1371/journal.pmed.1003646 (PMC8213195; doi:10.1371/journal.pmed.1003646)
Supplement: S2 Appendix — (DOCX) [file pmed.1003646.s002.docx]

Supplementary Appendix 2: Detailed Risk of Bias Assessments for Comparative Analyses

[1. ART initiation among HIV positive 2](#_Toc67127446)

[a. ART initiation among HIV positive: Randomized controlled trails 2](#_Toc67127447)

[b. ART initiation among HIV positive: Cohort studies 3](#_Toc67127448)

[2. Retention among HIV-positive at 12 months 4](#_Toc67127449)

[a. Retention among HIV-positive at 12 months: Randomized controlled trials 4](#_Toc67127450)

[b. Retention among HIV-positive at 12 months: cohorts 6](#_Toc67127451)

[3. Viral suppression among HIV-positive at 12 months 7](#_Toc67127452)

[a. Viral suppression among HIV-positive at 12 months: Randomized controlled trials 7](#_Toc67127453)

[b. Viral suppression among HIV-positive at 12 months: cohort studies 9](#_Toc67127454)

[4. Mortality 10](#_Toc67127455)

[a. Mortality: RCTS 10](#_Toc67127456)

[b. Mortality cohort 11](#_Toc67127457)

[1. Adherence 12](#_Toc67127458)

[a. Adherence: RCTS 12](#_Toc67127459)

[b. Adherence: Cohorts 13](#_Toc67127460)

## ART initiation among HIV positive

## ART initiation among HIV positive: Randomized controlled trails

| **Study** | **Sequence Generation** | **Allocation Concealment** | **Blinding: Participants & Personnel** | **Blinding: Outcome Assessment** | **Attrition Bias** | **Selective Reporting** | **Other Bias** | **Cluster: Recruitment Bias** | **Cluster: Baseline Imbalance** | **Cluster: Loss of clusters** | **Cluster: Analysis** | **Overall ROB** |
| --- | --- | --- | --- | --- | --- | --- | --- | --- | --- | --- | --- | --- |
| **Labhardt 2018** | A computer-generated randomization list was generated in block sizes of 4. Participants were randomly assigned (1:1 allocation) | Sealed, sequentially numbered, opaque envelopes were used to allocate participants | Although participants and personnel were unblinded - unlikely that this resulted in performance bias | Outcome assessors were not blinded. Measurement of outcome unlikely to be affected by detection bias. | Outcome was measured at the start of study. Unlikely to be affected by attrition bias. | Outcome was not reported as a specific outcome in the study but not affected by selective reporting |  |  |  |  |  | Low risk |
| **MacPherson 2014** | Random allocation through drawing colored balls out of bag | Method of concealment was drawing of colored balls from an opaque bag held above eye level to select the distribution of clusters and group allocation | Although participants and personnel were unblinded - unlikely that this resulted in performance bias | Outcome assessors were not blinded but outcome measurement unlikely to be affected by detection bias | Outcome was measured at the start of study. Unlikely to be affected by attrition bias. | Outcome pre-specified in registration. |  | Intervention was delivered after cluster randomization. Unlikely that participants would relocate to a new area to be able to get home ART initiation in the trial | Baseline characteristics were well balanced between groups | No clusters were lost to follow up | Not clearly specified in the Methods but ICC reported suggesting adjustment for clustering | Low risk |

## ART initiation among HIV positive: Observational studies

| **Study** | **Representativeness of the exposed cohort** | **Selection of the non-exposed cohort** | **Ascertainment of exposure** | **Demonstration that outcome of interest was not present at start of study** | **Comparability of cohorts on the basis of the design or analysis controlled for confounders** | **Assessment of outcome** | **Follow-up long enough for outcome to occur** | **Adequacy of follow-up of cohorts** | **Overall ROB** |
| --- | --- | --- | --- | --- | --- | --- | --- | --- | --- |
| **Oladele 2018*** | Ten LGAs that received the intervention from both rural and urban settings | Only 4 LGA's were selected for the control cohort - fundamental differences in the control group - much lower HIV incidence - these also were only urban | Secure record - Data was extracted from the Nigeria District Health Information System (DHIS) platform for LGA-based HIV services from all health facilities providing HIV services within all the study LGAs | Participants tested positive and had not been on ART before being assessed for the outcome | Unadjusted estimates used in review analysis - fundamental difference present in the control and comparison arms e.g. much lower HIV prevalence in the control clusters and all urban | Record linkage - National service register where outcome was assigned a code | Yes - Outcome occurred following testing | No statement - loss to follow up was not reported | Poor Quality |
| **Reif 2017** | Adolescents enrolled were ages 10-20 years at the GHESKIO Adolescent HIV Clinic | Different source - Intervention group historical cohort | Ascertainment of exposure not described | Participants were all newly tested HIV+ at baseline and were not on ART | Unadjusted estimates used in analysis; no comparison of baseline characteristics to evaluate | Record linkage - ART initiation determined by documentation of ART start in EMR | Yes - follow-up time was sufficient | The outcome of ART initiation was reported for all participants | Poor Quality |
| **Vu 2019** | Eligible participants were women who sold sex for money or goods in the past 6 months, aged 18+, HIV-positive, and not currently on ART | Different source - Intervention group was recruited from Njombe region and the comparison group was recruited from Mbeya region | Ascertainment of exposure was not described | Participants were FSW who were HIV positive and not on ART | Unadjusted estimates used in analysis; and there was baseline imbalance in the characteristics in the groups; intervention group had substantially more newly diagnosed participants uptake and retention | Self-report during 6 months post-enrollment interview | Yes - follow-up period was sufficient | <20% LTFU in each arm; non-differential ltfu - no difference in characteristics of ltfu compared to retained at 6 months. | Poor Quality |

*Although authors conducted an interrupted time series analysis which was more appropriate for accounting for temporal trends and baseline imbalances, these data could not be included in meta-analysis and there unadjusted raw data was included in the review

## Retention among HIV-positive at 12 months

## Retention among HIV-positive at 12 months: Randomized controlled trials

| **Study** | **Sequence Generation** | **Allocation Concealment** | **Blinding: Participants & Personnel** | **Blinding: Outcome Assessment** | **Attrition Bias** | **Selective Reporting** | **Other Bias** | **Cluster: Recruitment Bias** | **Cluster: Baseline Imbalance** | **Cluster: Loss of clusters** | **Cluster: Analysis** | **Overall ROB** |
| --- | --- | --- | --- | --- | --- | --- | --- | --- | --- | --- | --- | --- |
| **Amstutz 2021** | Computer generated list by independent statistician – randomized 1:1:1:1 block sizes of 4 | Allocation could not be concealed recruiting teams were aware of household assignments | Although participants and personnel were unblinded - unlikely that this resulted in performance bias | Not blinded however extensive tracing conducted for all study participants at 12 months | Not applicable for this outcome | None |  | Intervention was delivered after cluster randomization. unlikely that participants would relocate to a new area to be able to get VHW follow-up and SMS reminders | Some baseline imbalance of participant characteristics and number of participants likely due to chance | 2 clusters lost from control and 6 lost from intervention. However due to the high number of clusters (311) this is unlikely to bias outcomes | ICC reported and cluster adjusted analyses presented | Some concerns |
| **Labhardt 2018** | A computer-generated randomization list was generated in block sizes of 4. Participants were randomly asigned (1:1 allocation) | Sealed, sequentially numbered, opaque envelopes were used to allocate participants | Although participants and personnel were unblinded - unlikely that this resulted in performance bias | Unclear if outcome assessors were blinded. | Not applicable for this outcome | None |  |  |  |  |  | Low risk |
| **MacPherson 2014** | Random allocation through drawing coloured balls out of bag | Method of concealment was drawing of coloured balls from an opaque bag held above eye level to select the distribution of clusters and group allocation | Although participants and personnel were unblinded - unlikely that this resulted in performance bias | Data from clinic registers and treatment cards extracted without reference to group. Investigator blinding was maintained until the final analysis | Not applicable for this outcome | None |  | Intervention was delivered after cluster randomization. unlikely that participants would relocate to a new area to be able to get home ART initiation in the trial | Baseline characteristics were well balanced between groups | No clusters were lost to follow up | Not clearly specified in the Methods but ICC was reported and adjustment for clustering | Low risk |

##

## Retention among HIV-positive at 12 months: Observational studies

| **Study** | **Representativeness of the exposed cohort** | **Selection of the non-exposed cohort** | **Ascertainment of exposure** | **Demonstration that outcome of interest was not present at start of study** | **Comparability of cohorts on the basis of the design or analysis controlled for confounders** | **Assessment of outcome** | **Follow-up long enough for outcome to occur** | **Adequacy of follow-up of cohorts** | **Overall ROB** |
| --- | --- | --- | --- | --- | --- | --- | --- | --- | --- |
| **Reif 2017** | Adolescents enrolled were ages 10-20 years at the GHESKIO Adolescent HIV Clinic | Different source - Intervention group historical cohort | Ascertainment of exposure not described | Retention in care only relevant at follow-up | Unadjusted estimates used in analysis; no comparison of baseline characteristics to evaluate | Record linkage - Retention at 12 months was based on being alive with a clinic visit between 11 and 13 months from HIV testing. | Yes - follow-up time was sufficient | Retention in care described for both arms | Poor Quality |
| **Vu 2019** | Eligible participants were women who sold sex for money or goods in the past 6 months, aged 18+, HIV-positive, and not currently on ART | Different source - Intervention group was recruited from Njombe region and the comparison group was recruited from Mbeya region | Ascertainment of exposure was not described | Retention in care only relevant at follow-up | Unadjusted estimates used in analysis; and there was baseline imbalance in the characteristics in the groups; with the intervention group had substantially more newly diagnosed participant which could affect uptake and retention | Self report during 12 months post-enrollment interview; and differential outcome assessment | Yes - follow-up period was sufficient | Retention only assess for the few patients who got the interview question and this was differential between arms | Poor Quality |

## Viral suppression among HIV-positive at 12 months

## Viral suppression among HIV-positive at 12 months: Randomized controlled trials

| **Study** | **Sequence Generation** | **Allocation Concealment** | **Blinding of Participants and Personnel (Performance bias)** | **Blinding of Outcome Assessment (Detection bias)** | **Attrition Bias** | **Selective Reporting** | **Other Bias** | **Cluster: Recruitment Bias** | **Cluster: Baseline Imbalance** | **Cluster: Loss of clusters** | **Cluster: Analysis** | **Overall ROB** |
| --- | --- | --- | --- | --- | --- | --- | --- | --- | --- | --- | --- | --- |
| **Amstutz 2021** | Computer generated list by independent statistician – randomized 1:1:1:1 block sizes of 4 | Allocation could not be concealed recruiting teams were aware of household assignments | Although participants and personnel were unblinded - unlikely that this resulted in performance bias | Laboratory staff at health facility conducted VL for all participants and were unaware of assignment | Outcome included only those with both 6 and 12 month study visit. 13 in control and 9 in intervention who did not have a viral load - considered as failures. | None |  | Intervention was delivered after cluster randomization. unlikely that participants would relocate to a new area to be able to get VHW follow-up and SMS reminders | Some baseline imbalance of participant characteristics and number of participants but likely due to chance | 2 clusters lost from control and 6 lost from intervention. However due to the high number of clusters (311) this is unlikely to bias outcomes | ICC reported and cluster adjusted analyses presented | Some concerns |
| **Labhardt 2018** | A computer-generated randomization list was generated in block sizes of 4. Participants were randomly assigned (1:1 allocation) | Sealed, sequentially numbered, opaque envelopes were used to allocate participants | Although participants and personnel were unblinded - unlikely that this resulted in performance bias | Laboratory staff at health facility conducted VL for all participants and were unaware of assignment | Loss to follow-up was 8.8% in the same day group and 7.3% in the usual care group | Reported outcome specified in the protocol |  |  |  |  |  | Low risk |
| **Barnabas 2020** | The study biostatistician generated the randomisation allocation stratified by site and country.  Participants in the same household  were randomised to the same group to prevent crossover between study groups. | The randomisation allocation was automatically assigned by the mobile phone software to the study  participant. Once eligibility was assessed, the randomisation assignment was revealed. The study  staff did not have access to the randomisation code. | Although participants and personnel were unblinded - unlikely that this resulted in performance bias | Laboratory staff, who assessed  the primary outcome of plasma HIV viral load, were blinded to the allocation of participants as  were the study investigators. | 71/1531 LTFU < 5% | Although other outcomes like uptake and retention were not reported – viral load was their primary outcome and is reported in the main paper |  |  |  |  |  | Low risk |

| **Study** | **Representativeness of the exposed cohort** | **Selection of the non-exposed cohort** | **Ascertainment of exposure** | **Demonstration that outcome of interest was not present at start of study** | **Comparability of cohorts on the basis of the design or analysis controlled for confounders** | **Assessment of outcome** | **Follow-up long enough for outcome to occur** | **Adequacy of follow-up of cohorts** | **Overall ROB** |
| --- | --- | --- | --- | --- | --- | --- | --- | --- | --- |
| **Vu 2019** | Eligible participants were women who sold sex for money or goods in the past 6 months, aged 18+, HIV-positive, and not currently on ART | Intervention group was recruited from Njombe region and the comparison group was recruited from Mbeya region | Ascertainment of exposure was not described | Participants were FSW who were HIV positive and not on ART | Unadjusted estimates used in analysis; and there was baseline imbalance in the characteristics in the groups; the intervention group had substantially more newly diagnosed participant which could affect uptake and retention | Clinic/study records | 12 months likely sufficient | Lost-to-follow-up participants (15%) were comparable across the groups | Poor Quality |

## Viral suppression among HIV-positive at 12 months: Observational studies

## Mortality

## Mortality at any time point: Randomized controlled trials

| **Study** | **Sequence Generation** | **Allocation Concealment** | **Blinding: Participants & Personnel** | **Blinding: Outcome Assessment** | **Attrition Bias** | **Selective Reporting** | **Other Bias** | **Cluster: Recruitment Bias** | **Cluster: Baseline Imbalance** | **Cluster: Loss of clusters** | **Cluster: Analysis** | **Overall ROB** |
| --- | --- | --- | --- | --- | --- | --- | --- | --- | --- | --- | --- | --- |
| Amstutz 2021 | Computer generated list by independent statistician – randomized 1:1:1:1 block sizes of 4 | Allocation could not be concealed recruiting teams were aware of household assignments | Although participants and personnel were unblinded - unlikely that this resulted in performance bias | Unlikely that mortality assessment was differential – extensive tracing undertaken to determine outcome at 12 months in both arms | 4% LTFU at 12 months in both arms | None |  | Intervention was delivered after cluster randomization. unlikely that participants would relocate to a new area to be able to get VHW follow-up and SMS reminders | Some baseline imbalance of participant characteristics and number of participants but likely due to chance | 2 clusters lost from control and 6 lost from intervention. However due to the high number of clusters (311) this is unlikely to bias outcomes | No cluster adjustment, too few events | Some concerns |
| Labhardt  2018 | A computer-generated randomization list was generated in block sizes of 4. | Sealed, sequentially numbered, opaque envelopes | Although participants and personnel were unblinded - unlikely that this resulted in performance bias | Unlikely that outcome assessment was differential – extensive tracing undertaken to determine outcome at 12 months | LTFU <10% in both groups |  |  |  |  |  |  | Low risk |
| MacPherson 2014 | Random allocation through drawing coloured balls out of bag | Drawing of coloured balls from an opaque bag held above eye level to select the distribution of clusters and group allocation | Although participants and personnel were unblinded - unlikely that this resulted in performance bias | Treatment records were used to determine participants who died Investigator blinding was maintained | Loss from ART over 6 months 28.7% in home group; 23.8% in facility group |  |  | No participants were recruited after cluster assignment | Baseline characteristics were well balanced between the groups | No reported loss of cluster | Cluster adjusted | Some concerns |

## Mortality: Observational studies

| **Study** | **Representativeness of the exposed cohort** | **Selection of the non-exposed cohort** | **Ascertainment of exposure** | **Demonstration that outcome of interest was not present at start of study** | **Comparability of cohorts on the basis of the design or analysis controlled for confounders** | **Assessment of outcome** | **Follow-up long enough for outcome to occur** | **Adequacy of follow-up of cohorts** | **Overall ROB** |
| --- | --- | --- | --- | --- | --- | --- | --- | --- | --- |
| Vu 2019 | Eligible participants were women who sold sex for money or goods in the past 6 months, aged 18+, HIV-positive, and not currently on ART | Different source - Intervention group was recruited from Njombe region and the comparison group was recruited from Mbeya region | Ascertainment of exposure was not described | Pariticipants were alive at the start of the study | Unadjusted data used in the analysis | Confirmed by Sauti CBHTC and clinical team as well as the NIMR Regional Research Coordinator | 6 months likely sufficient | Similar lost-to-follow-up participants across the groups | Poor Quality |

## Adherence

## Adherence: Randomized controlled trials

| **Study** | **Sequence Generation** | **Allocation Concealment** | **Blinding: Participants & Personnel** | **Blinding: Outcome Assessment** | **Attrition Bias** | **Selective Reporting** | **Other Bias** | **Cluster: Recruitment Bias** | **Cluster: Baseline Imbalance** | **Cluster: Loss of clusters** | **Cluster: Analysis** | **Overall ROB** |
| --- | --- | --- | --- | --- | --- | --- | --- | --- | --- | --- | --- | --- |
| MacPherson 2014 | Random allocation through drawing coloured balls out of bag | drawing of coloured balls from an opaque bag held above eye level | Although participants and personnel were unblinded - unlikely that this resulted in performance bias | Outcome assessment was unblinded; self-reported adherence was assessed by questionnaire using the AIDS Clinical Trials Group adherence questionnaire | LTFU at 6 months was 28.7% in the home group and 23.8% in the facility group | Outcome was pre-specified in registration |  | No participants were recruited after cluster assignment | Baseline well balanced between the groups except for reported household deaths in the previous year | No clusters were reported as lost | Result suggests adjustment for clustering was done | High risk |

## Adherence: Observational studies

| **Study** | **Representativeness of the exposed cohort** | **Selection of the non-exposed cohort** | **Ascertainment of exposure** | **Demonstration that outcome of interest was not present at start of study** | **Comparability of cohorts on the basis of the design or analysis controlled for confounders** | **Assessment of outcome** | **Follow-up long enough for outcome to occur** | **Adequacy of follow-up of cohorts** | **Overall ROB** |
| --- | --- | --- | --- | --- | --- | --- | --- | --- | --- |
| Vu 2019 | Eligible participants were women who sold sex for money or goods in the past 6 months, aged 18+, HIV-positive, and not currently on ART | Different source - Intervention group was recruited from Njombe region and the comparison group was recruited from Mbeya region | Ascertainment of exposure was not described | Participants were FSW who were HIV positive and not on ART | Unadjusted estimates used in analysis; and there was baseline imbalance in the characteristics in the groups; with the intervention group had substantially more newly diagnosed participant which could affect uptake and retention | Self-report during 6 months post-enrollment interview | 6 months likely sufficient | Similar lost-to-follow-up participants across the groups | Poor quality |
