# Supplementary figures and images for: Effects of community-based antiretroviral therapy initiation models on HIV treatment outcomes: A systematic review and meta-analysis
Source: PLoS Med. 2021 May 28;18(5):e1003646. doi: 10.1371/journal.pmed.1003646 (PMC8213195; doi:10.1371/journal.pmed.1003646)

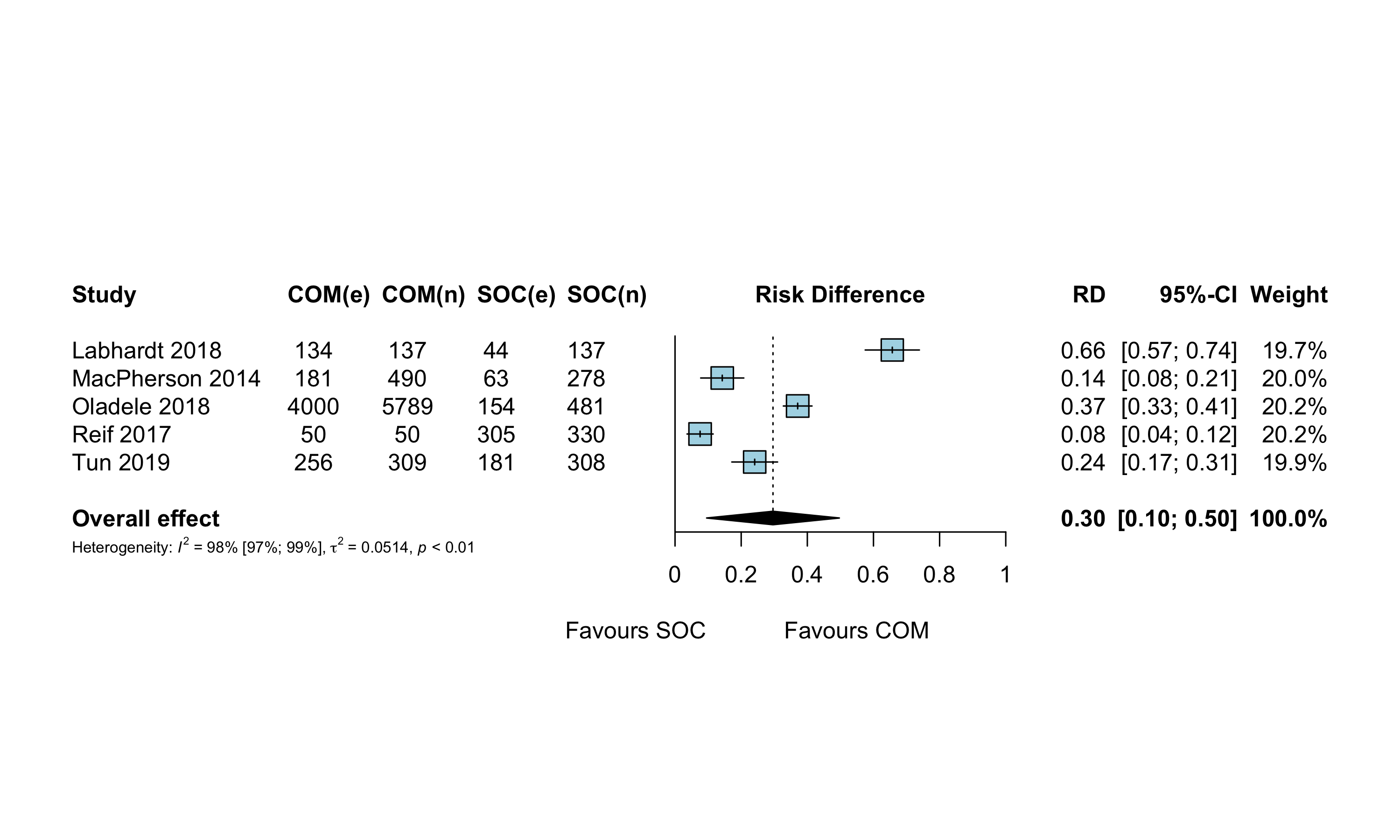

Supplement: S1 Fig — (PNG) [file pmed.1003646.s003.png]

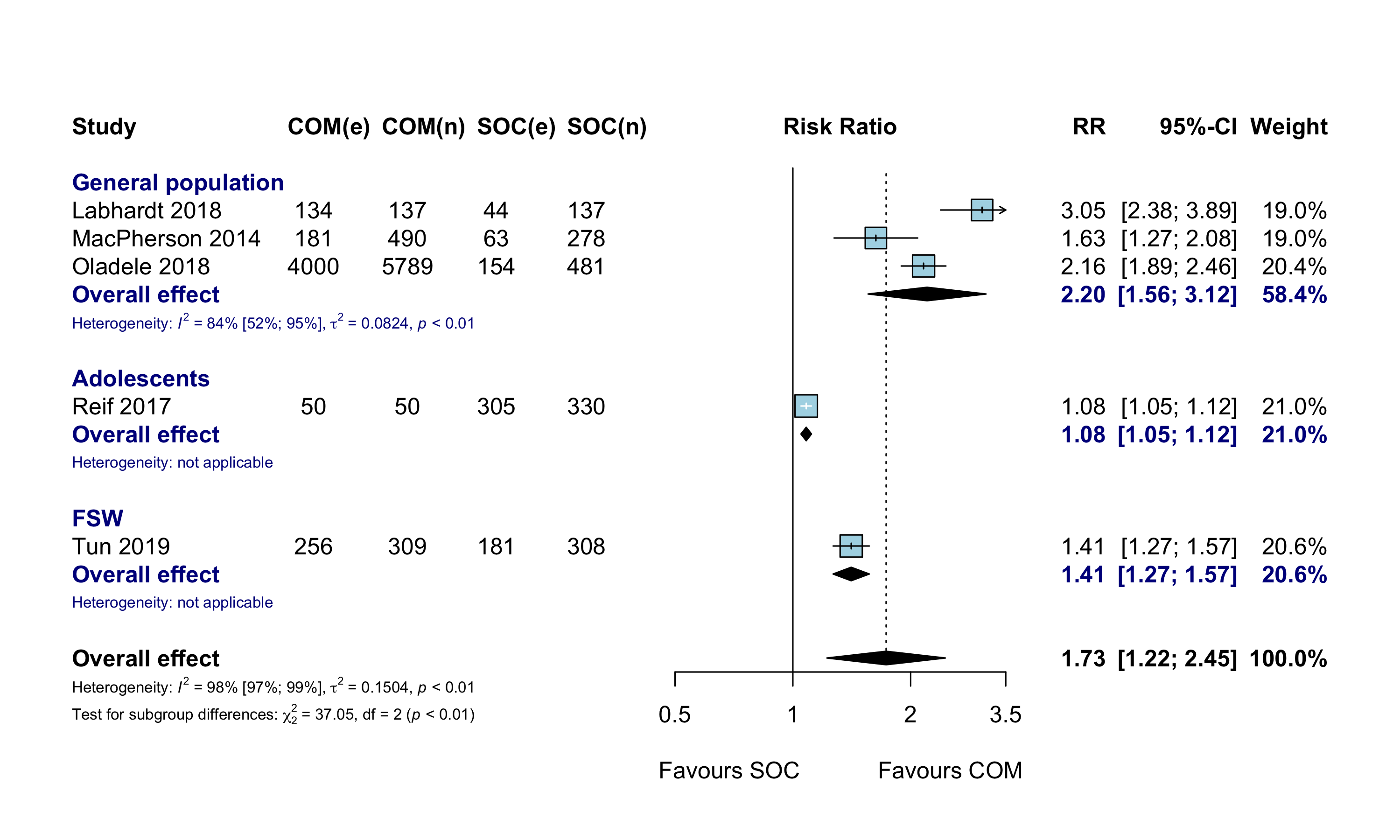

Supplement: S2 Fig — (PNG) [file pmed.1003646.s004.png]

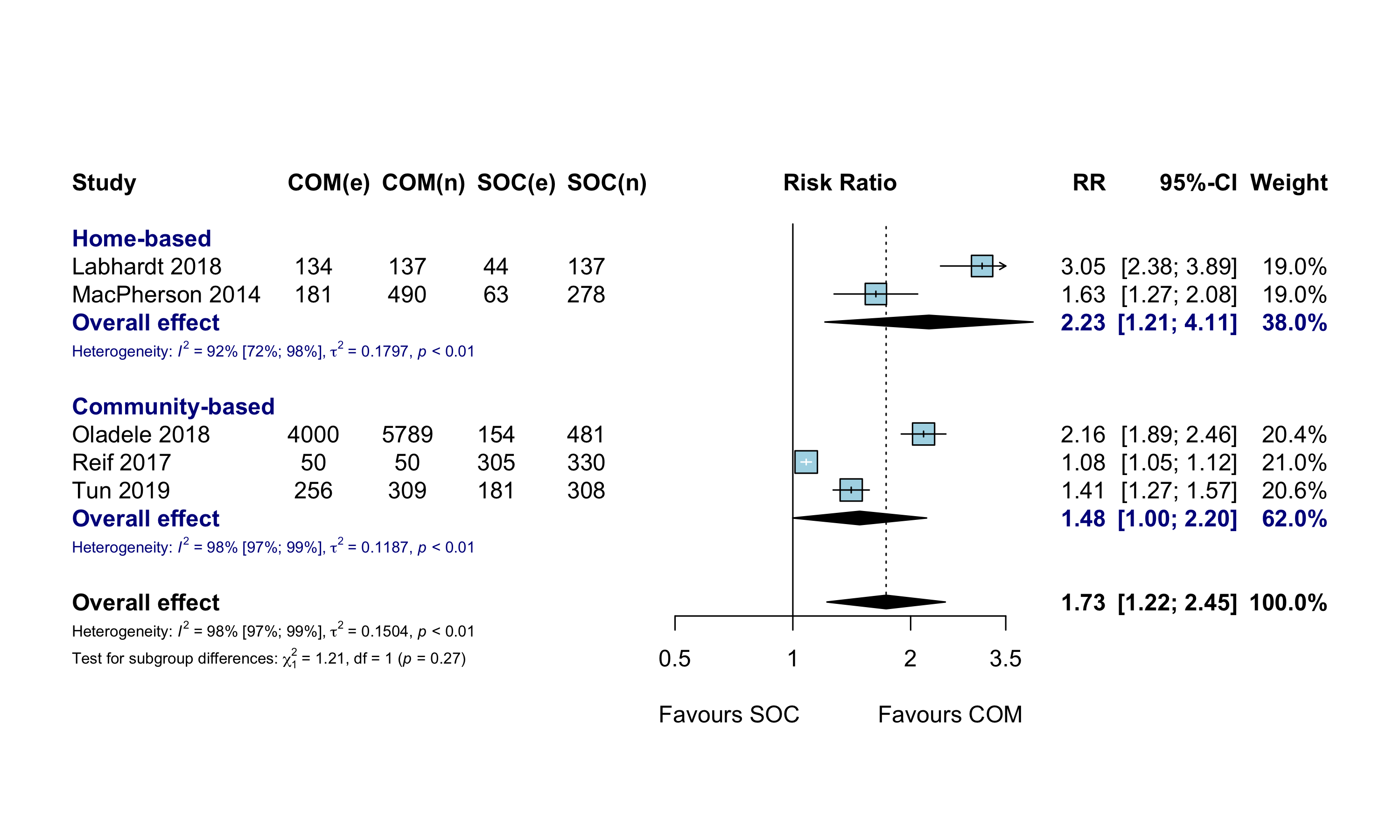

Supplement: S3 Fig — (PNG) [file pmed.1003646.s005.png]

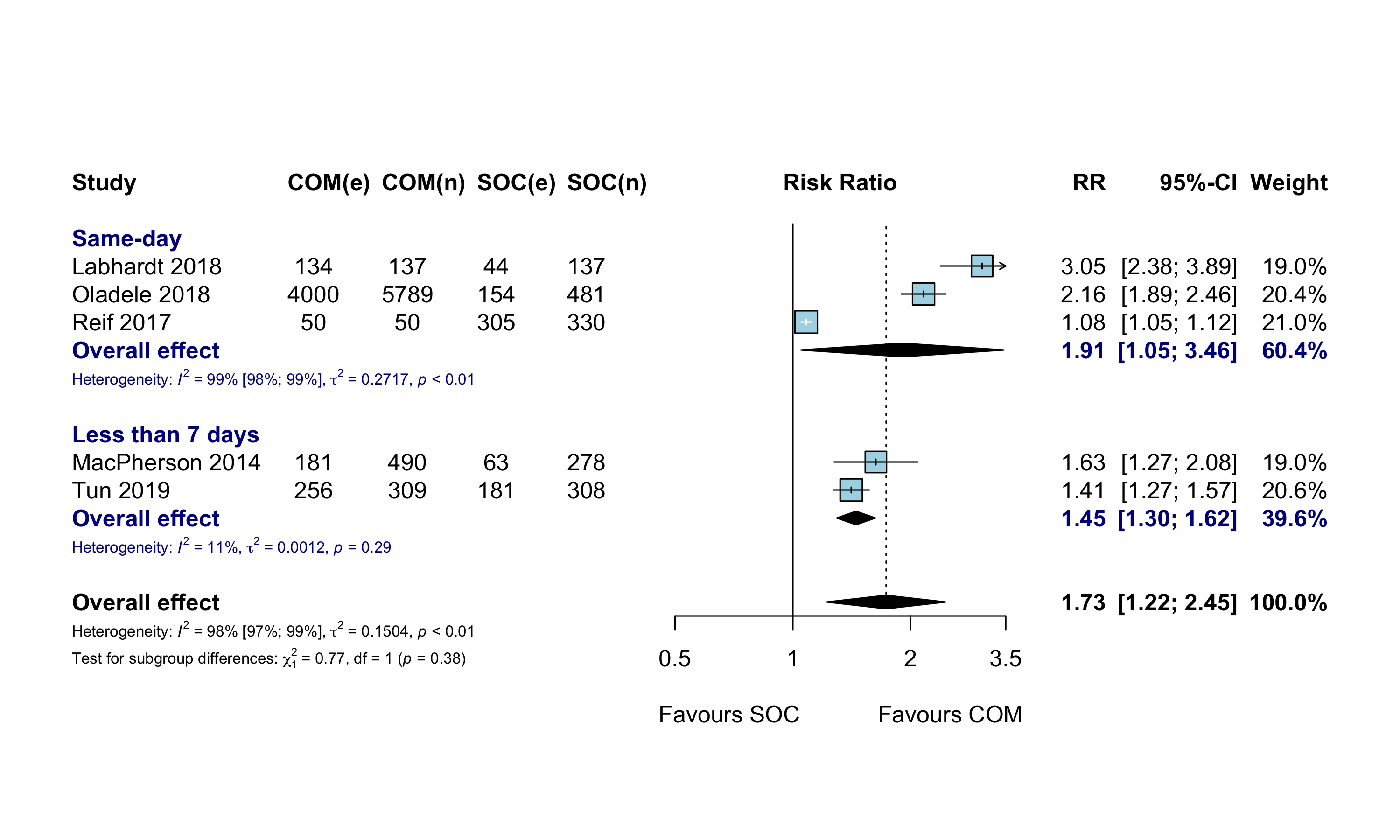

Supplement: S4 Fig — (PNG) [file pmed.1003646.s006.png]

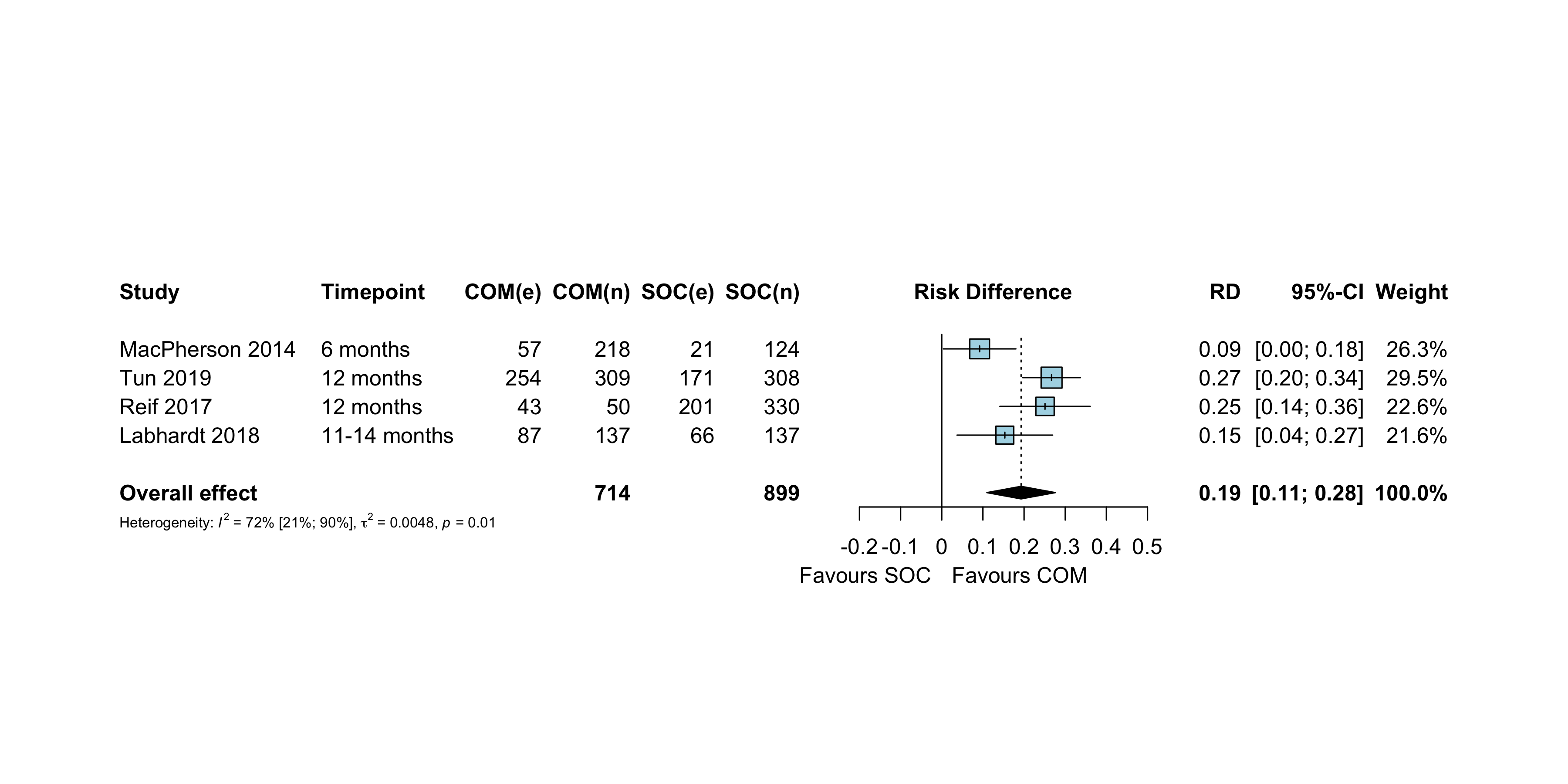

Supplement: S5 Fig — (PNG) [file pmed.1003646.s007.png]

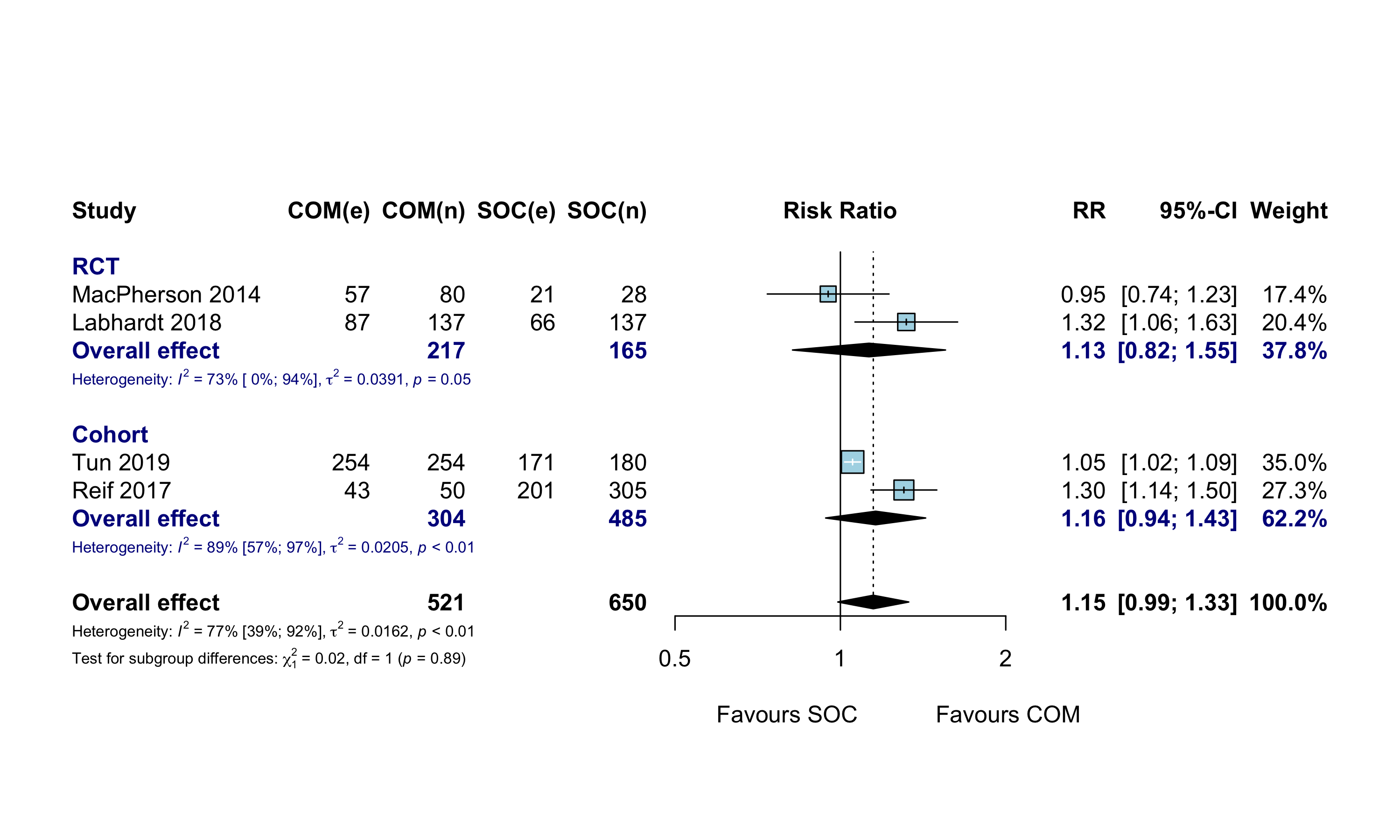

Supplement: S6 Fig — (PNG) [file pmed.1003646.s008.png]

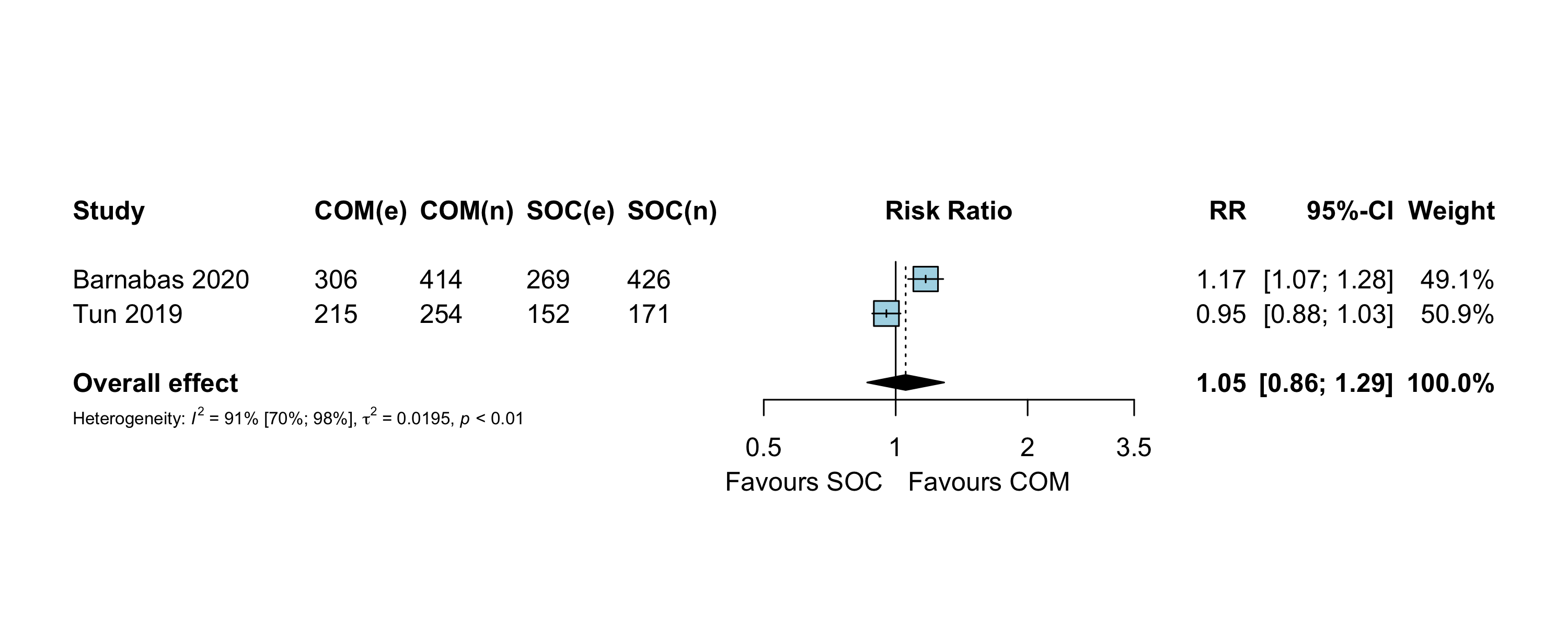

Supplement: S7 Fig — (PNG) [file pmed.1003646.s009.png]
