# Supplementary material for: Effects of community-based antiretroviral therapy initiation models on HIV treatment outcomes: A systematic review and meta-analysis
Source: PLoS Med. 2021 May 28;18(5):e1003646. doi: 10.1371/journal.pmed.1003646 (PMC8213195; doi:10.1371/journal.pmed.1003646)
Supplement: S2 Table — (DOCX) [file pmed.1003646.s011.docx]

**S2 Table: Detailed PRECIS-2 criteria**

| **Study** | **Eligibility** | | **Recruitment** | | **Setting** | | **Organization** | | **Flexibility in delivery of intervention** | | **Flexibility in adherence to intervention** | | **Follow-up** | | **Primary Outcome** | | **Primary Analysis** | |
| --- | --- | --- | --- | --- | --- | --- | --- | --- | --- | --- | --- | --- | --- | --- | --- | --- | --- | --- |
| **Barnabas 2020** | 4 | Excluded CD4 <100. Establishing CD4 in the community may be challenging in real world setting. | 4 | Recruited during HIV testing which was part of the study –at home or mobile van, trading posts and in the evenings. | 5 | Embedded in the community | 3 | 1 Nurse + Lay Counsellor – food parcels as provided at each visit in both arms; incentives provided | 4 | Flexibility implicit in design, ART initiation procedures similar to routine care, but trial setting likely results in greater protocol adherence | 5 | No additional measures to ensure adherence | 5 | Routine follow-up in both arms | 5 | The primary outcome is directly relevant to the participants | - | Unclear conference |
| **MacPherson 2014** | 5 | Those eligible represent those eligible in a real-world setting. | 4 | Recruitment approximates what may occur in a real-world setting | 5 | Community Health worker catchment areas | 3 | Possible that trial staff were trained better/had more resources than real world setting | 3 | Not described but given community-based nature likely flexible | 5 | No additional measures to improve adherence | 4 | No more than routine follow-up was done | 5 | The primary outcome is directly relevant to the participants | 5 | Intention to treat - efficacy |
| **Labhardt 2018** | 4 | Several exclusions: pregnancy, breastfeeding, advanced disease, CRAG positive, any chronic disease | 3 | This size of the recruitment team may not feasible in the routine setting. | 5 | Care was provided at the routine health facility or in the home | 3 | Teams were employed by the trial which may involve a different level of training to what may occur in usual care | 4 | This approximates what may occur in a routine setting. | 4 | No additional measures to improve adherence | 2 | Possible that the intensity of tracing was greater than routine care | 5 | The primary outcome is directly relevant to the participants | 5 | Intention to treat - efficacy |
| **Amstutz 2021** | 5 | Eligibility represents those from real world setting with the exception of exclusion of hypertensives and diabetics | 4 | Study recruitment team consisting of study nurse, counsellors and campaign manager, appears similar to what may be feasible in a routine setting | 5 | Rural villages in Northern Lesotho, care provided in homes or VHW home or routine health facility | 3 | Teams were employed by the trial which may involve a different level of training to what may occur in usual care; targeted SMS delivery dependent on well functioning information systems | 4 | Flexibility implicit in design, ART initiation procedures similar to routine care, but trial setting likely results in greater protocol adherence | 5 | No additional measures to ensure adherence | 4 | To obtain trial outcomes follow-up may be more intensive than routine care | 5 | The primary outcome is directly relevant to the participants | 5 | Cluster adjusted and intent to treat |
| **Vu 2019** | 5 | Those eligible represent those from a real-world setting | 3 | Recruitment was nested within routine services and approximates what may occur outside of a research study | 5 | Real world setting for FSW | 3 | Care was nested within routine services; but $4.5 incentives per visit | 4 | Flexibiity in delivery to accommodate the population group | 5 | Approximates usual care | 3 | Possible that follow-up was more intensive in the intervention arm; participants also received $4.5 incentives per visit to improve follow-up | 5 | The primary outcome is directly relevant to the participants | 5 | Intention to treat - efficacy |
| **Reif 2017** | 5 | Those eligible represent those from a real-world setting | 5 | Recruitment was nested within routine services | 5 | Routine adolescent services or community room | 4 | This level of care may be feasible in routine care | 5 | Routine care provided in one arm and community care likely requiring a great level of flexibility | 5 | Adherence support similar to what would occur in real world setting for both groups | 4 | Follow-up likely approximates what would occur in routine services | 5 | The primary outcome is directly relevant to the participants | 3 | Analysis conducted for all who entered the program |
| **Oladele 2018** | 5 | Eligibility criteria for ART was per national guideline. | 5 | Large community programs; real-world approach | 3 | PEPFAR supported regions | 3 | PEPFAR supported large outreach team, may not be feasible without donor support | 5 | Task-sharing: between providers with community lay workers. | 5 | Flexibility essential in order to deliver the intervention | 5 | Routine follow-up at health facility after ART initiation | 5 | Relevant outcomes | 4 | Slightly unclear what the denominator was for several analysis |
| **Ibiloye 2018** | 5 | The KPs that received the community-based HIV care likely represent KP's in the region | 4 | This is similar to the usual care | 5 | Outreach venues were areas within the community where KP's would routinely be accessed | 3 | Relatively large outreach team, may not be feasible outside of a well funded program | 5 | Flexibility implicit in design and given population group | 5 | Flexibility essential in order to deliver the interventions | 3 | Immediate tracing of lost patients - may be better than usual care | 5 | Relevant outcomes | 5 | Intention to treat - efficacy |
