# Supplementary material for: Effects of community-based antiretroviral therapy initiation models on HIV treatment outcomes: A systematic review and meta-analysis
Source: PLoS Med. 2021 May 28;18(5):e1003646. doi: 10.1371/journal.pmed.1003646 (PMC8213195; doi:10.1371/journal.pmed.1003646)
Supplement: S3 Table — (DOCX) [file pmed.1003646.s012.docx]

**S3 Table: Ongoing studies**

| **Study ID** | **Study title** | **Country** | **Setting** | **Participants** | **Expected completion** | **Clinicaltrials.gov** |
| --- | --- | --- | --- | --- | --- | --- |
| **Gadjah Mada 2018 (34)** | HIV Awal (Early) Testing & Treatment Indonesia Project Intervention Phase | Indonesia | NR | ≥16 years; 1000 HIV+ | December 31, 2019 | https://clinicaltrials.gov/ct2/show/NCT03659253 |
| **Ferrand 2022; NCT03719521 (35)** | Community Based Interventions to Improve HIV Outcomes in Youth: a Cluster Randomised Trial in Zimbabwe | Zimbabwe | NR | 16 to 24 years; 24000 HIV+ | September 30, 2022 | https://clinicaltrials.gov/ct2/show/NCT03719521 |
| **Iveth 2019; NCT01792752 2013 (36)** | Enhanced Access to HIV Care for Drug Users in San Juan, Puerto Rico | Puerto Rico | NR | ≥18 years; 4110 HIV+ | August 2020 | https://clinicaltrials.gov/ct2/show/NCT01792752 |
